# Performance of prediction models for delayed union and nonunion after fracture: a systematic review and meta-analysis

**DOI:** 10.1186/s12891-026-09941-4

**Published:** 2026-05-29

**Authors:** Fengsheng Yin, Xiangjin Wang, Gan Luo, Zhen Deng, Wentao Chen, Yu Zhang

**Affiliations:** 1 Department of Orthopedics, Chengdu Integrated TCM & Western Medicine Hospital, Chengdu University of Traditional Chinese Medicine, Chengdu, 610041 Sichuan China; 2 School of Clinical Medicine, Chengdu University of Traditional Chinese Medicine, Chengdu, 610075 Sichuan China; 3 School of Acupuncture and Massage Therapy, Chengdu University of Traditional Chinese Medicine, Chengdu, Sichuan 610075 China

**Keywords:** Delayed union, Nonunion, Fracture healing, Prediction model, Systematic review, Meta-analysis

## Abstract

**Background:**

Delayed union and nonunion are common and costly complications after fractures, yet early risk stratification remains challenging. We systematically reviewed and meta-analyzed multivariable prediction models for compromised fracture healing.

**Methods:**

MEDLINE, EMBASE, CINAHL, SinoMed, and CNKI were searched from inception to 30 November 2025. Studies developing or validating models predicting delayed union, nonunion, or related healing outcomes after fractures were included. Risk of bias was assessed with PROBAST and certainty of evidence with GRADE. Discrimination was summarized using the area under the receiver operating characteristic curve (AUC), pooled with random-effects models by validation tier (apparent performance, internal validation, external validation), and further described by anatomical subgroup. The protocol was registered in PROSPERO (CRD420251252244).

**Results:**

Seventy-seven studies reporting 97 model entries were included across appendicular, proximal femoral, multisite, and axial skeletal fracture settings; 56 models reported apparent performance only, 28 had internal validation, and 13 underwent external validation. Pooled AUC was 0.88 (95% CI 0.86–0.90) for apparent performance, 0.72 (95% CI 0.41–0.90) for internal validation, and 0.81 (95% CI 0.73–0.86) for external validation, with substantial heterogeneity. Apparent performance exceeded validated performance (mean optimism 0.052).

**Conclusion:**

Existing models often show optimistic apparent discrimination; however, discrimination alone is insufficient to justify clinical implementation, especially given limited external validation, sparse calibration reporting, major clinical and anatomical heterogeneity, and high risk of bias. Future studies should prioritize clearer outcome definitions, robust methodology, transparent reporting, independent external validation with calibration assessment, and evaluation of clinical utility before these models are used to guide care.

**Supplementary Information:**

The online version contains supplementary material available at 10.1186/s12891-026-09941-4.

## Background

Fractures are among the most common injuries worldwide and impose substantial economic and societal burdens [1]. Although most fractures heal uneventfully, a clinically meaningful minority progress to delayed union or nonunion, with reported rates ranging from 1.9% to 4.9% [2, 3]. The interpretation of these estimates is limited by the lack of consensus definitions. Nonunion is generally defined as a fracture that is unlikely to unite without further intervention; however, the diagnostic time threshold varies widely across studies (approximately 3–12 months after injury) [4, 5]. Furthermore, definitions of delayed union are even less consistent. These complications occur across fracture sites and patterns [3] and are associated with higher healthcare utilization, greater costs, and worse quality of life [6–9].

Delayed union and nonunion are often accompanied by persistent pain, functional limitations, and prolonged care [6]. They can result in repeated clinical visits and imaging, time away from work, and additional surgical procedures, increasing both direct and indirect costs [8]. Clinically, the key challenge is not confirming an established nonunion but rather identifying, early in follow-up, patients who are unlikely to achieve union with standard care while management remains modifiable. Early risk stratification could support closer surveillance and timely optimization of mechanical conditions and modifiable patient factors [10]. In the absence of reliable prognostic tools, these decisions are frequently made with substantial uncertainty [11].

Fracture repair reflects the interplay between biological and mechanical determinants shaped by host characteristics, injury severity, and treatment factors. Because these determinants vary across patients and settings, fractures treated in similar ways may still follow markedly different healing trajectories [12]. Heterogeneity is further amplified by variable outcome definitions and diagnostic time windows for delayed union and nonunion [5], limiting consistent outcome ascertainment across studies and clinical practice. Consequently, informal clinical judgment alone may be insufficiently reliable and may not be generalizable across settings [13].

Prediction models integrate multidimensional data (e.g., demographics, injury characteristics, and biomarkers) to estimate an individual’s risk of compromised fracture healing [14]. Prognostic modeling has expanded rapidly and now includes regression-based, score-based, and machine-learning approaches [15, 16], yet readiness for clinical use remains uncertain. The evidence base is limited by methodological heterogeneity, particularly inconsistent outcome definitions and time windows [5], as well as small sample sizes that increase the risk of overfitting [17]. Moreover, external validation and calibration are often absent or incompletely reported, limiting confidence in performance in new populations [18]. Therefore, a systematic synthesis is needed to appraise both performance and methodological quality.

We conducted a systematic review and meta-analysis of prediction models for delayed union and nonunion. We aimed to synthesize predictive performance, prioritize externally validated results when available, and identify methodological gaps that may inform the development and validation of more robust and clinically useful tools for predicting compromised fracture healing.

## Methods

### Study design and registration

This systematic review and meta-analysis was conducted and reported in accordance with the Preferred Reporting Items for Systematic Reviews and Meta-Analyses (PRISMA) statement [19]. The protocol was prospectively registered with PROSPERO (CRD420251252244).

### Search strategy

A medical librarian designed and executed search strategies in five databases—MEDLINE (Ovid), EMBASE (Elsevier), CINAHL (EBSCOhost), SinoMed, and CNKI—from inception to November 30, 2025. The searches used controlled vocabulary (e.g., MeSH terms) and free-text terms for compromised fracture healing (delayed union and nonunion) and clinical prediction modeling (e.g., prediction or prognosis, including regression-based and machine learning approaches). The full search strategies are provided in the supplementary information files.

### Eligibility criteria

We included studies of human participants with clinically or radiographically confirmed fractures without restrictions by fracture site, age, or treatment strategy. Eligible studies developed, updated, or validated multivariable prediction models (≥ 2 predictors) to estimate the individual risk of delayed union, nonunion, or re-nonunion; studies using a clearly defined composite “healing failure” endpoint were also eligible. Searches were not restricted by language; however, the full-text eligibility assessment was limited to English or Chinese for feasibility.

We excluded studies that (1) were not based on fracture-healing populations or patient-level clinical data; (2) did not report a fracture-healing endpoint; (3) did not present a multivariable prediction model; (4) predicted outcomes other than delayed union, nonunion, re-nonunion, or a clearly defined composite healing-failure endpoint; (5) were not primary research articles (e.g., reviews, guidelines, editorials, letters, or conference abstracts); and (6) lacked accessible full text or sufficient detail to identify the prediction model.

### Study selection and data collection process

Two reviewers independently screened titles and abstracts using Rayyan [20], followed by full-text eligibility assessment. Disagreements were resolved by discussion or, when needed, adjudicated by a third reviewer.

Two reviewers independently extracted data using a standardized form, with discrepancies resolved by consensus. We collected data on study, participant, and fracture characteristics, outcome definitions and assessment time points, and details of model development and validation. Performance metrics were extracted separately for development (apparent) and validation datasets, including discrimination (C-statistic/AUC) and any reported calibration information.

### Quality assessment and certainty of evidence

The risk of bias and applicability were assessed using PROBAST [21], which evaluates four domains (participants, predictors, outcome, and analysis). The domains were judged individually and then combined to determine the overall risk of bias. Applicability was assessed for participants, predictors, and outcome domains. Assessments were performed independently by two reviewers, and disagreements were resolved by consensus or third-party adjudication.

We used GRADE (Grading of Recommendations Assessment, Development and Evaluation) to rate certainty in the overall body of evidence [22], considering the risk of bias, inconsistency, imprecision, indirectness, and publication bias.

### Statistical analyses

The results were summarized narratively, and the predictors retained in the final models were mapped using a heatmap. Discrimination was assessed using the area under the receiver operating characteristic curve (AUC; c-statistic) and meta-analyzed separately for apparent performance (development), internal validation, and external validation; outcome-stratified analyses and descriptive anatomical subgroup summaries were undertaken when feasible [29].

AUCs were pooled on a logit scale. When necessary, the confidence limits were truncated to 0.001 and 0.999 before the transformation to avoid infinite values. Standard errors were derived from reported 95% confidence intervals using a normal approximation on the logit scale, or estimated using the Hanley–McNeil method when confidence intervals were unavailable but sample size and event and non-event counts were reported [30]. Given the expected heterogeneity in reported performance, random-effects models were fitted using restricted maximum likelihood with Hartung–Knapp–Sidik–Jonkman confidence intervals as a conservative specification for heterogeneous meta-analytic data [23, 24]. Heterogeneity was summarized using I² and τ², and prediction intervals were reported wherever feasible. When studies contributed multiple eligible estimates, we fitted multilevel random-effects models with study-level cluster-robust variance estimation [25].

Where sufficient data were available, we conducted exploratory subgroup analyses and meta-regression. For nonunion models, the meta-regression included eligible performance estimates across validation tiers, with validation tier entered as a moderator. Sensitivity analyses examined the influence of individual studies and key analytical choices. Small-study effects were assessed using funnel plots and Egger regression when at least ten studies were available [26]. Analyses were conducted using the R software (version 4.3.3).

## Results

### Study selection

**Fig. 1:**
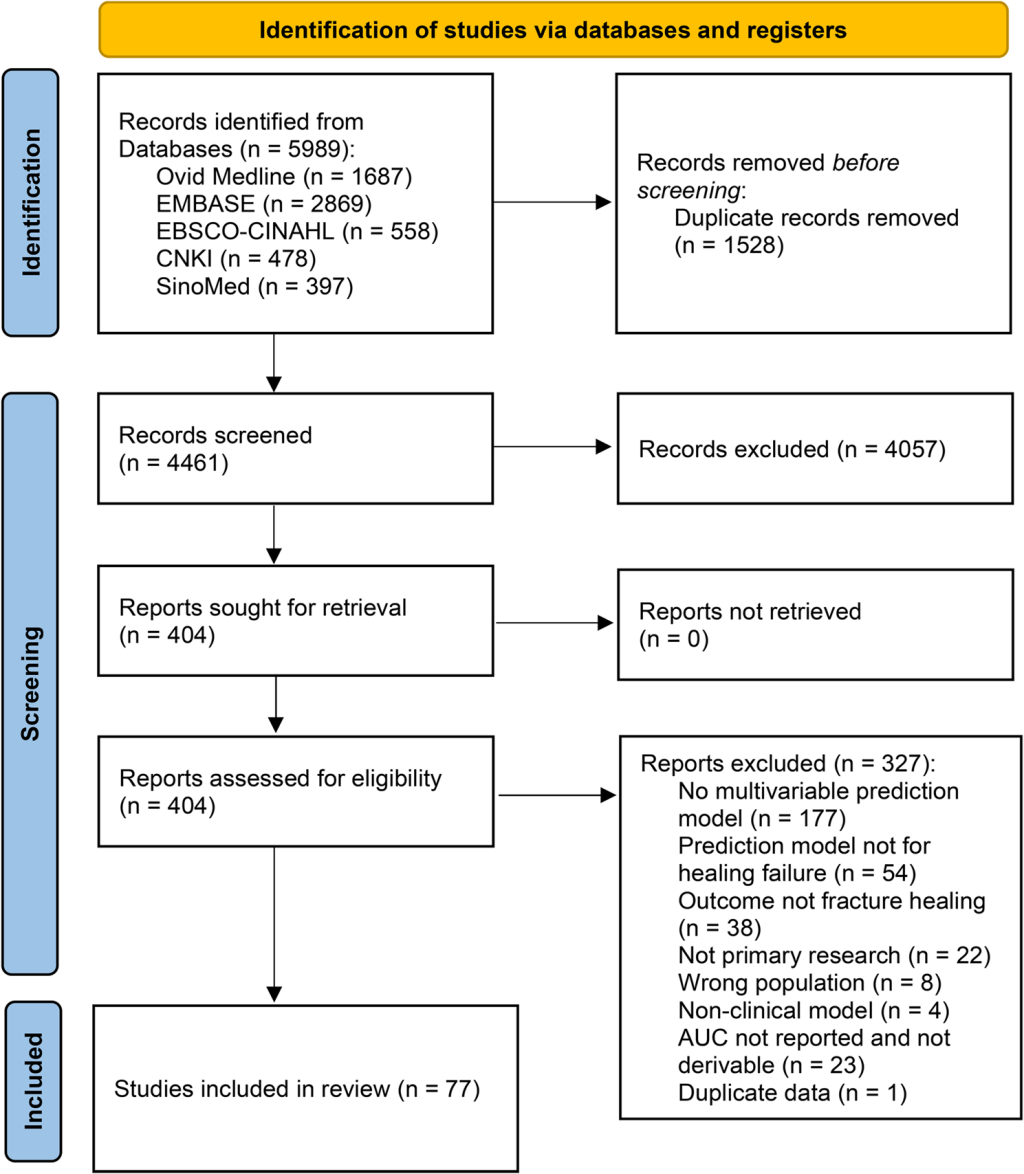
PRISMA flow diagram of the study selection process

The database searches identified 5,989 records. After removing 1,528 duplicate records, 4,461 unique records were retained for title and abstract screening. At this stage, 4,057 articles were excluded, leaving 404 articles for full-text assessment. Following full-text review, 327 articles were excluded, and 77 studies met the eligibility criteria and were included in the systematic review and meta-analysis. The study selection process is summarized in the PRISMA flow diagram (Fig. 1).

### Study characteristics

Seventy-seven studies were included (Table 1). Most studies were conducted in China (*n* = 56, 73%) [27–82], followed by the UK (*n* = 5, 6%) [83–87] and the USA (*n* = 4, 5%) [88–91]. The remaining studies were conducted in Germany (*n* = 2) [92, 93], Italy (*n* = 2) [94, 95], the Netherlands (*n* = 1) [96], Japan (*n* = 1) [97], Taiwan (*n* = 1) [98], Finland (*n* = 1) [99], and multinational collaborations (*n* = 4) [100–103]. Retrospective cohort studies predominated (*n* = 62; 81%; Table 1). Six studies were prospective cohort studies [34, 54, 58, 85, 94, 98], six were case–control studies [27, 47, 62, 72, 77, 103], and three used other designs (two database studies [90, 91] and one secondary analysis of an RCT [102]).

**Table 1:** Characteristics of included studies and prediction models

Study (Author, Year)	Country	Study design	Setting / data source	Population / fracture site	Treatment	Sample size (*N*)	Outcome	Prediction horizon	Outcome ascertainment	Predictors summary	Model / algorithm	Validation tier	Discrimination (AUC)
Shen et al., 2025	China	Retrospective cohort; Single-center	Dept of Orthopedics, General hospital inpatients; Single hospital records (development: Jul 2020–Apr 2023; validation: May 2023–Jan 2024)	Spine; Osteoporotic fractures (spine, hip, proximal humerus, distal radius); baseline: Acute/stress fracture	Unclear/Mixed	316	Nonunion	> 6-12mo	Clinical+Imaging	Imaging/Radiographic; Clinical/Demographic; vars 5	logistic regression; logistic regression (nomogram)	External	0.83
Chen et al., 2024	China	Retrospective cohort; Single-center	Dept of Orthopedics, General hospital inpatients; Single hospital records (Jan 2014–Jul 2021)	Lower limb; Osteoporotic fractures (hip, proximal femur); baseline: Acute/stress fracture	Unclear/Mixed	226	Nonunion	> 6-12mo	Clinical+Imaging	Imaging/Radiographic; vars 5	logistic regression	Internal	0.81
Chun et al., 2020	China	Retrospective cohort; Single-center	Dept of Orthopedics, General hospital inpatients; Single hospital records (Jul 2015–Dec 2018)	Lower limb; Femoral shaft fracture in elderly patients treated with internal fixation (high- and low-energy trauma); baseline: Acute/stress fracture	Surgical	188–241	Nonunion	> 6-12mo	Radiograph	Clinical/Demographic; Omics/miRNA; vars 6	logistic regression; logistic regression (nomogram)	External	0.77
Deng et al., 2019	China	Retrospective cohort; Single-center	Dept of Orthopedics, General hospital inpatients; Single hospital records (Jan 2015–Jan 2017; follow-up to Jan 2018)	Lower limb; Tibial fracture treated with intramedullary nail fixation; baseline: Acute/stress fracture	Surgical	71	Nonunion	> 6-12mo	Clinical+Imaging	Serum/Blood biomarkers; vars 3	logistic regression	Apparent	0.79
Guo et al., 2024	China	Retrospective cohort; Single-center	Dept of Orthopedics, General hospital inpatients; Single hospital records (Jun 2019–Apr 2022)	Upper limb; Distal radius fracture treated with volar plate open reduction and internal fixation (traumatic); baseline: Acute/stress fracture	Surgical	94	Delayed union	<=3mo	Clinical+Imaging	Imaging/Radiographic; vars 5	logistic regression	Apparent	0.84
Guo et al., 2023	China	Retrospective cohort; Single-center	Dept of Orthopedics, General hospital inpatients; Single hospital records (Jan 2018–Dec 2021)	Other; Extremity fractures treated surgically (traumatic); baseline: Acute/stress fracture	Surgical	188	Delayed union	> 3-6mo	Clinical+Imaging	Omics/miRNA; vars 2	logistic regression	Apparent	0.94
Guo et al., 2024	China	Retrospective cohort; Single-center	Dept of Orthopedics, General hospital inpatients; Single hospital records (Jan 2020–Jan 2022)	Other; Extremity fractures (traumatic); baseline: Acute/stress fracture	Unclear/Mixed	140	Delayed union	<=3mo	Clinical+Imaging	Omics/miRNA; vars 2	logistic regression	Apparent	0.86
Hao et al., 2024	China	Retrospective cohort; Single-center	Dept of Orthopedics, General hospital inpatients; Single hospital records (Sep 2022–Aug 2023)	Other; Long bone fractures of extremities (traumatic); baseline: Acute/stress fracture	Unclear/Mixed	156	Delayed union	<=3mo	Clinical+Imaging	Serum/Blood biomarkers; vars 2	logistic regression	Apparent	0.86
He et al., 2024	China	Retrospective cohort; Single-center	Dept of Orthopedics, General hospital inpatients; Single hospital records (Jan 2019–Dec 2023)	Lower limb; Intertrochanteric femoral fractures treated with PFNA (traumatic); baseline: Acute/stress fracture	Surgical	112	Poor/non-healing	<=3mo	Clinical+Imaging	Serum/Blood biomarkers; vars 3	logistic regression	Apparent	0.80
Huo et al., 2024	China	Prospective cohort; Single-center	Dept of Orthopedics, General hospital inpatients; Single hospital records (May 2020–May 2023)	Multi-site; Limb fractures treated surgically (multi-site (femur, tibia, femoral neck, humerus, radius), traumatic); baseline: Acute/stress fracture	Surgical	292	Delayed union	> 3-6mo	Clinical+Imaging	Imaging/Radiographic; vars 3	NR	Apparent	0.94
JIANG et al., 2025	China	Retrospective cohort; Single-center	Dept of Orthopedics, General hospital inpatients; Single hospital records (Mar 2022–Mar 2024)	Other; Osteoporotic spinal fractures treated with PVP; baseline: Acute/stress fracture	Surgical	139	Delayed union	<=3mo	Clinical+Imaging	Serum/Blood biomarkers; vars 2	NR	Apparent	0.88
Jin et al., 2025	China	Retrospective cohort; Single-center	Dept of Orthopedics, General hospital inpatients; Single hospital records (Jan 2021–Nov 2023)	Lower limb; Femoral neck fracture after surgery (traumatic); baseline: Acute/stress fracture	Surgical	201	Delayed union	<=3mo	Clinical+Imaging	Serum/Blood biomarkers; vars 2	NR	Apparent	0.86
Liang et al., 2024	China	Retrospective cohort; Single-center	Dept of Orthopedics, General hospital inpatients; Single hospital records (Apr 2020–Apr 2022)	Lower limb; Femoral shaft fracture treated with interlocking intramedullary nailing (traumatic); baseline: Acute/stress fracture	Surgical	133	Nonunion	> 6-12mo	Clinical+Imaging	Serum/Blood biomarkers; vars 2	combined index (combined index of ODF and IGF-1; method NR)	Apparent	0.91
Liao et al., 2024	China	Retrospective cohort; Single-center	Dept of Orthopedics, General hospital inpatients; Single hospital records (Jan 2022–Dec 2023)	Multi-site; Traumatic fractures after surgery (upper and lower limb fractures); baseline: Acute/stress fracture	Surgical	108	Delayed union	> 6-12mo	Clinical+Imaging	Serum/Blood biomarkers; vars 3	combined index (combined index of P1NP, OST and β-CTx; method NR)	Apparent	0.86
Liu et al., 2025	China	Retrospective cohort; Single-center	Dept of Orthopedics, General hospital inpatients; Single hospital records (May 2021–May 2024)	Other; Diaphyseal fractures of extremities treated surgically with intramedullary nailing (traumatic); baseline: Acute/stress fracture	Surgical	152	Delayed union	<=3mo	Clinical+Imaging	Serum/Blood biomarkers; vars 2	logistic regression	Apparent	0.93
Liu et al., 2023	China	Retrospective cohort; Single-center	Dept of Orthopedics, General hospital inpatients; Single hospital records (Feb 2020–Feb 2023)	Lower limb; Tibial fracture (traumatic); baseline: Acute/stress fracture	Unclear/Mixed	122	Delayed union	> 3-6mo	Radiograph	Serum/Blood biomarkers; vars 2	combined index (combined index of β-CTX and TPINP; method NR)	Apparent	0.90
Liu et al., 2025	China	Retrospective cohort; Single-center	Dept of Orthopedics, General hospital inpatients; Single hospital records (Sep 2023–Sep 2024)	Craniofacial; Traumatic (mandibular condylar fractures treated with open reduction and internal fixation); baseline: Acute/stress fracture	Surgical	31	Poor/non-healing	> 3-6mo	Clinical+Imaging	Serum/Blood biomarkers; vars 2	logistic regression	Apparent	0.96
Ma et al., 2023	China	Retrospective cohort; Single-center	Dept of Orthopedics, General hospital inpatients; Single hospital records (Jan 2015–Dec 2021)	Lower limb; Tibial plateau fractures treated with internal fixation (traumatic); baseline: Acute/stress fracture	Surgical	342	Nonunion	> 6-12mo	Clinical+Imaging	Imaging/Radiographic; vars 9	logistic regression	Internal	0.89
Qian et al., 2022	China	Retrospective cohort; Single-center	Dept of Orthopedics, General hospital inpatients; Single hospital records (Jan 2019–Jan 2021)	Lower limb; Tibial fractures treated with open reduction and interlocking intramedullary nailing (traumatic); baseline: Acute/stress fracture	Surgical	90	Delayed union	> 3-6mo	Clinical+Imaging	Serum/Blood biomarkers; vars 3	logistic regression	Apparent	0.91
REN et al., 2022	China	Retrospective cohort; Single-center	Dept of Orthopedics, General hospital inpatients; Single hospital records (Feb 2019—Jun 2021)	Lower limb; Tibial plateau fractures treated with internal fixation; baseline: Acute/stress fracture	Surgical	129	Delayed union	<=3mo	Radiograph	Omics/miRNA; vars 2	logistic regression	Apparent	0.92
Su et al., 2022	China	Retrospective cohort; Single-center	Dept of Orthopedics, General hospital inpatients; Single hospital records (Feb 2019–Jun 2021)	Lower limb; Femoral neck fractures (traumatic, treated with internal fixation); baseline: Acute/stress fracture	Surgical	251	Delayed union	<=3mo	CT/CBCT	Omics/miRNA; vars 2	logistic regression	Apparent	0.92
WANG et al., 2022	China	Retrospective cohort; Single-center	Dept of Orthopedics, General hospital inpatients; Single hospital records (Jan 2020–Mar 2021)	Lower limb; Intertrochanteric femoral fractures (treated with internal fixation); baseline: Acute/stress fracture	Surgical	140	Delayed union	<=3mo	CT/CBCT	Serum/Blood biomarkers; vars 2	logistic regression	Apparent	0.82
WANG et al., 2024	China	Retrospective cohort; Single-center	Dept of Orthopedics, General hospital inpatients; Single hospital records (Jan 2020–Apr 2023)	Spine; Osteoporotic vertebral compression fractures (treated with PVP or PKP); baseline: Acute/stress fracture	Surgical	260	Delayed union	<=3mo	Clinical+Imaging	Serum/Blood biomarkers; vars 2	logistic regression	Apparent	0.86
WANG et al., 2021	China	Retrospective cohort; Single-center	Dept of Orthopedics, General hospital inpatients; Single hospital records (Jun 2013–Jun 2020)	Lower limb; Femoral shaft fractures (closed, middle third); baseline: Acute/stress fracture	Unclear/Mixed	183	Nonunion	> 6-12mo	Clinical+Imaging	Omics/miRNA; vars 4	logistic regression	Internal	0.95
WANG et al., 2024	China	Case-control; Single-center	Dept of Orthopedics, General hospital inpatients; Single hospital records (Jan 2022–Mar 2023)	Multi-site; Traumatic fractures (various sites, treated with internal fixation); baseline: Acute/stress fracture	Surgical	142	Delayed union	<=3mo	Radiograph	Serum/Blood biomarkers; vars 3	logistic regression	Apparent	0.93
Xu et al., 2021	China	Retrospective cohort; Single-center	Dept of Orthopedics, General hospital inpatients; Single hospital records (Aug 2019–Aug 2020)	Lower limb; Tibial plateau fractures (traumatic, treated with arthroscopy-assisted open reduction and internal fixation); baseline: Acute/stress fracture	Surgical	178	Delayed union	> 6-12mo	Clinical+Imaging	Serum/Blood biomarkers; vars 3	logistic regression	Apparent	0.86
Yang et al., 2020	China	Retrospective cohort; Single-center	Dept of Orthopedics, General hospital inpatients; Single hospital records (Oct 2017–Nov 2018)	Lower limb; Tibial fractures (traumatic, treated with intramedullary nail fixation); baseline: Acute/stress fracture	Surgical	80	Healing composite	> 6-12mo	Clinical+Imaging	Serum/Blood biomarkers; vars 3	logistic regression	Apparent	0.91
Yang et al., 2024	China	Retrospective cohort; Single-center	Dept of Orthopedics, General hospital inpatients; Single hospital records (Jun 2020–Dec 2023)	Multi-site; Tibial plateau and shaft fractures (mixed trauma); baseline: Acute/stress fracture	Surgical	150	Delayed union	<=3mo	Radiograph	Omics/miRNA; vars 2	logistic regression	Apparent	0.88
Ye et al., 2023	China	Retrospective cohort; Single-center	Dept of Orthopedics, General hospital inpatients; Single hospital records (May 2018–May 2021)	Lower limb; Closed tibial shaft fractures (traumatic, treated surgically); baseline: Acute/stress fracture	Surgical	136	Delayed union	> 6-12mo	Clinical+Imaging	Omics/miRNA; vars 2	logistic regression	Apparent	0.81
Yuan et al., 2024	China	Case-control; Single-center	Dept of Orthopedics, General hospital inpatients; Single hospital records (Jan 2020–Jun 2023)	Lower limb; Ankle fractures with triangular ligament injury (traumatic; surgically treated); baseline: Acute/stress fracture	Surgical	490	Delayed union	<=3mo	Radiograph	Omics/miRNA; vars 13	logistic regression (nomogram)	Apparent	0.87
Zhang et al., 2023	China	Retrospective cohort; Single-center	Dept of Orthopedics, General hospital inpatients; Single hospital records (Jan 2019–Jun 2021)	Spine; Osteoporotic vertebral compression fractures (elderly diabetic, treated with percutaneous vertebroplasty); baseline: Acute/stress fracture	Surgical	108	Delayed union	<=3mo	Clinical+Imaging	Serum/Blood biomarkers; vars 2	logistic regression	Apparent	0.77
Zhang et al., 2022	China	Retrospective cohort; Single-center	Dept of Orthopedics, General hospital inpatients; Single hospital records (Jan 2019–Jan 2021)	Lower limb; Femoral shaft fractures (elderly, traumatic, treated with compression plate internal fixation); baseline: Acute/stress fracture	Surgical	112	Nonunion	> 6-12mo	Clinical+Imaging	Serum/Blood biomarkers; vars 2	logistic regression	Apparent	0.91
Zhang et al., 2024	China	Retrospective cohort; Multi-center	Dept of Orthopedics, General hospital inpatients; Two-hospital records (Jan 2022–Jul 2023)	Lower limb; Femoral fractures (traumatic, treated with internal fixation); baseline: Acute/stress fracture	Surgical	104	Delayed union	NR	Clinical+Imaging	Serum/Blood biomarkers; vars 3	logistic regression	Apparent	0.97
Zhao et al., 2023	China	Case-control; Single-center	Dept of Orthopedics, General hospital inpatients; Single hospital records (Jan 2018–Jul 2022)	Lower limb; Femoral fractures (treated with intramedullary nail fixation); baseline: Acute/stress fracture	Surgical	140	Delayed union	> 3-6mo	CT/CBCT	Serum/Blood biomarkers; vars 2	logistic regression	Apparent	0.87
Zheng et al., 2024	China	Retrospective cohort; Single-center	Dept of Orthopedics, General hospital inpatients; Single hospital records (Jan 2020–Feb 2022)	Lower limb; Femoral neck fractures (treated with internal fixation); baseline: Acute/stress fracture	Surgical	173	Healing composite	> 6-12mo	Clinical+Imaging	Serum/Blood biomarkers; vars 4	logistic regression	Apparent	0.92
Zhou et al., 2024	China	Retrospective cohort; Single-center	Dept of Orthopedics, General hospital inpatients; Single hospital records (Jan 2020–Oct 2022)	Lower limb; Femoral neck fractures (fresh traumatic, treated with internal fixation); baseline: Acute/stress fracture	Surgical	292	Delayed union	> 3-6mo	Clinical+Imaging	Omics/miRNA; vars 3	logistic regression	Apparent	0.84
WANG et al., 2025	China	Retrospective cohort; Single-center	Dept of Orthopedics, General hospital inpatients; Single hospital records (Jan 2022–Jun 2024)	Lower limb; Tibial and fibular fractures (traumatic, treated surgically); baseline: Acute/stress fracture	Surgical	246	Delayed union	> 6-12mo	Clinical+Imaging; Radiograph	Serum/Blood biomarkers; vars 6	logistic regression	Internal	0.71
Manokian et al., 2025	Netherlands	Retrospective cohort; Single-center	Dept of Orthopedics, General hospital inpatients; Single hospital records (Jan 2012–May 2024)	Upper limb; Displaced midshaft clavicle fractures (traumatic, treated nonoperatively); baseline: Acute/stress fracture	Nonoperative	374	Nonunion	> 3-6mo	Clinical+Imaging	Imaging/Radiographic; vars 3	logistic regression	Internal	0.70
Tang et al., 2025	China	Retrospective cohort; Single-center	Dept of Orthopedics, General hospital inpatients; Single hospital CT imaging database (2012–2023)	Rib; Rib fractures (traumatic, non-operatively managed); baseline: Acute/stress fracture	Nonoperative	297	Poor/non-healing	> 3-6mo	CT/CBCT	Imaging/Radiographic; Clinical/Demographic; vars 2–4	logistic regression	Internal	0.49–0.70
Braun et al.,2025	Germany	Retrospective cohort; Single-center	Dept of Orthopedics, General hospital inpatients; Single hospital nonunion database (continuous enrollment starting Feb 2009)	Lower limb; Distal femur nonunions (post-traumatic; surgically treated); baseline: Established nonunion/defect	Surgical	45	Treatment/Management outcome	> 3-6mo	Surgery event	Imaging/Radiographic; vars 8	scoring system (LEG NUI)	External	0.76
Gamada et al., 2024	Japan	Retrospective cohort; Single-center	Dept of Orthopedics, General hospital inpatients; Single hospital records (Apr 2014–Mar 2023)	Spine; Lumbar spondylolysis (stress fractures in adolescent athletes); baseline: Acute/stress fracture	Unclear/Mixed	416	Nonunion	> 3-6mo	CT/CBCT	Clinical/Demographic; vars 2	risk score	Internal	0.85
Song et al., 2025	China	Prospective cohort; Single-center	Dept of Orthopedics, General hospital inpatients; Single hospital records (Jun 2022–Jun 2024)	Spine; Osteoporotic vertebral compression fractures (fragility, treated with percutaneous vertebroplasty); baseline: Acute/stress fracture	Surgical	300	Delayed union	<=3mo	Radiograph	Omics/miRNA; vars 5	logistic regression	Internal	0.87
ZHU et al., 2025	China	Retrospective cohort; Single-center	Dept of Orthopedics, General hospital inpatients; Single hospital records and stored serum samples (timeframe NR)	Spine; Osteoporotic fractures (ribs, upper limb, vertebral; fragility fractures treated conservatively with fixation); baseline: Acute/stress fracture	Nonoperative	51	Healing composite	<=3mo	Clinical+Imaging	Omics/miRNA; vars 2	logistic regression	Apparent	0.90
Wu et al., 2025	China	Retrospective cohort; Single-center	Dept of Orthopedics, General hospital inpatients; Single hospital records (Mar 2021–Dec 2024)	Lower limb; Intertrochanteric femoral fractures (elderly, treated with internal fixation); baseline: Acute/stress fracture	Surgical	889	Poor/non-healing	<=3mo	Radiograph	Omics/miRNA; vars 6	logistic regression	Apparent	0.95
Xiao et al., 2024	China	Retrospective cohort; Single-center	Dept of Orthopedics, General hospital inpatients; Single hospital records (Jun 2021–Jun 2023)	Spine; Thoracolumbar osteoporotic vertebral compression fractures (fragility); baseline: Acute/stress fracture	Unclear/Mixed	139	Delayed union	> 3-6mo	Clinical+Imaging	Serum/Blood biomarkers; vars 2	logistic regression	Apparent	0.88
Leister et al., 2023	Multi-country (Austria, Germany)	Retrospective cohort; Multi-center	Dept of Orthopedics, General hospital inpatients; Multicenter routinely collected hospital records from four tertiary referral centers (years NR)	Spine; Type II and III odontoid fractures (cervical spine fractures in adults, mostly elderly); baseline: Acute/stress fracture	Unclear/Mixed	415	Nonunion	> 3-6mo	CT/CBCT	Imaging/Radiographic; vars 7	logistic regression	Internal	0.71
Han et al., 2025	China	Retrospective cohort; Single-center	Dept of Orthopedics, General hospital inpatients; Single spinal center hospital records (Mar 2016–Mar 2021)	Spine; Osteoporotic vertebral compression fractures (single-segment thoracolumbar OVCFs, low-energy fragility fractures); baseline: Acute/stress fracture	Unclear/Mixed	208	Nonunion	> 3-6mo	MRI	Imaging/Radiographic; vars 4	risk score	Apparent	0.90
Hsu et al., 2016	Taiwan, China	Retrospective cohort; Single-center	Dept of Orthopedics, General hospital inpatients; Single level I trauma center records (Jan 2000–Jun 2014)	Lower limb; Intertrochanteric fractures (AO/OTA 31-A1 and A2, age ≥ 60 years, treated with DHS); baseline: Acute/stress fracture	Surgical	442	Treatment/Management outcome	<=3mo	Clinical+Imaging	Imaging/Radiographic; vars 4	risk score	Apparent	0.85
Haubruck et al., 2018	Germany	Prospective cohort; Single-center	Dept of Orthopedics, General hospital inpatients; Single university hospital long-bone non-union program records with prospective chemokine sampling (Mar 2012–Mar 2014)	Lower limb; Femoral and tibial diaphyseal non-unions (lower limb long-bone non-unions treated with Masquelet technique); baseline: Established nonunion/defect	Surgical	20	Treatment/Management outcome	> 6-12mo	Clinical+Imaging	Serum/Blood biomarkers; vars 2	logistic regression	Apparent	0.92
Kraus et al., 2024	USA	Retrospective cohort; Multi-center	Dept of Orthopedics, General hospital inpatients; Trauma registries and hospital records from two Level I trauma centers (2014–2020)	Lower limb; Femoral shaft fractures (AO/OTA 32 A–C) treated with reamed, statically locked intramedullary nailing (traumatic fractures); baseline: Acute/stress fracture	Surgical	305	Nonunion	> 12-24mo	Clinical+Imaging	Imaging/Radiographic; vars 6	risk score (FeNNR score)	Apparent	0.77
Li et al., 2024	China	Retrospective cohort; Single-center	Dept of Orthopedics, General hospital inpatients; Single hospital records (Jan 2015–Dec 2021)	Lower limb; Closed femoral shaft fractures (diaphyseal; AO/OTA 32-A/B/C; predominantly high-energy traumatic injuries treated operatively); baseline: Acute/stress fracture	Surgical; Unclear/Mixed	617	Healing composite; Nonunion	> 6–12mo	Clinical+Imaging; Radiograph	Imaging/Radiographic; Clinical/Demographic; vars 4	logistic regression	External	0.78
Suter et al., 2024	Finland	Retrospective cohort; Multi-center	Dept of Orthopedics, General hospital inpatients; Single tertiary hospital retrospective cohort (Jan 2006–Dec 2016) plus FISH randomized trial dataset (2012–2018)	Upper limb; Humeral shaft fractures (non-pathological, non-periprosthetic; treated nonoperatively); baseline: Acute/stress fracture	Nonoperative	226	Nonunion	<=3mo	Radiograph	Imaging/Radiographic; vars 4	scoring system	External	0.83–0.89
Panteli et al., 2021	UK	Retrospective cohort; Single-center	Dept of Orthopedics, General hospital inpatients; Single Level I trauma center records (Jan 2009–Dec 2016)	Lower limb; Subtrochanteric femur fractures (proximal femur; low-energy fragility and high-energy trauma treated with long intramedullary nailing); baseline: Acute/stress fracture	Surgical	316	Nonunion	> 6-12mo	Clinical+Imaging	Imaging/Radiographic; vars 7	logistic regression	Apparent	0.79
Duan et al., 2025	China	Retrospective cohort; Single-center	Dept of Orthopedics, General hospital inpatients; Single hospital records (Mar 2021–Nov 2024)	Multi-site; Open extremity fractures (upper and lower limb open fractures in elderly patients); baseline: Acute/stress fracture	Unclear/Mixed	221	Delayed union	> 3-6mo	CT/CBCT; Radiograph	Serum/Blood biomarkers; vars 7	logistic regression (nomogram)	External	0.95
WANG et al., 2021	China	Retrospective cohort; Multi-center	Dept of Orthopedics, General hospital inpatients; Multicenter hospital records (Feb 2014–Jan 2018)	Lower limb; Subtrochanteric femur fractures (intramedullary nailing; mostly low-energy fragility hip fractures in elderly with some high-energy trauma); baseline: Acute/stress fracture	Surgical	149	Nonunion	> 6-12mo	Clinical+Imaging	Imaging/Radiographic; vars 4	logistic regression	External	0.88
Zura et al., 2017	USA	Database study; Multi-center	Nationwide Medicare beneficiaries (hospital and outpatient fracture care); Medicare 5% Standard Analytic Files, USA (2011–2012)	Multi-site; Fractures at 18 skeletal sites (neck of femur, femur, tibia, fibula, tibia+fibula, humerus, radius, ulna, radius+ulna, pelvis, clavicle, ribs/trunk, patella, tarsal, metatarsal, metacarpal, scaphoid, ankle; mostly low-energy fragility fractures in older adults with some higher-energy trauma); baseline: Acute/stress fracture	Unclear/Mixed	56,492	Nonunion	> 6-12mo	Claims	Imaging/Radiographic; Clinical/Demographic; vars 26	logistic regression	External	0.71
Murray et al., 2013	UK	Retrospective cohort; Single-center	Dept of Orthopedics, General hospital inpatients; Single trauma unit records (Jan 1994–Dec 2007)	Upper limb; Displaced midshaft (diaphyseal) clavicle fractures (adult traumatic fractures treated nonoperatively); baseline: Acute/stress fracture	Nonoperative	941	Nonunion	> 3-6mo	Clinical+Imaging	Imaging/Radiographic; vars 3	logistic regression	Internal	0.85
ZHU et al., 2021	China	Retrospective cohort; Single-center	Dept of Orthopedics, General hospital inpatients; Single hospital records (Jan 2016–Jan 2019)	Lower limb; Nondisplaced femoral neck fractures (elderly; non-pathological; intracapsular); baseline: Acute/stress fracture	Unclear/Mixed	255	Treatment/Management outcome	> 24mo	Surgery event	Serum/Blood biomarkers; vars 6	logistic regression (nomogram)	Internal	0.81
Massari et al., 2018	Italy	Prospective cohort; Multi-center	Dept of Orthopedics, General hospital inpatients; multicenter hospital records (Jan 2010–Sep 2012)	Lower limb; Post-traumatic tibial fractures (AO types 41-A/B, 42-A/B/C, 43-A/B) treated surgically; baseline: Acute/stress fracture	Surgical	363	Nonunion	> 6-12mo	Clinical+Imaging	Omics/miRNA; vars 20	risk score (FRACTING score)	External	0.82
O’Hara et al., 2020	Multi-country (USA, Canada and other countries)	RCT secondary analysis; Multi-center	Dept of Orthopedics, General hospital inpatients; Single Level I trauma center trauma registry (derivation 2007–2014) and multicenter RCT dataset SPRINT trial (2000–2005)	Lower limb; Tibial shaft fractures treated with intramedullary nailing (reamed or unreamed); baseline: Acute/stress fracture	Surgical	382	Nonunion	> 6-12mo	Surgery event	Imaging/Radiographic; vars 9	logistic regression	External	0.61
Nicholson et al., 2020	UK	Prospective cohort; Single-center	Dept of Orthopedics, General hospital inpatients; Single university hospital trauma unit records (2-year recruitment period; exact dates NR)	Upper limb; Displaced midshaft clavicle fractures (Edinburgh type-2B, fully displaced); baseline: Acute/stress fracture	Unclear/Mixed	200	Nonunion	> 3-6mo	Clinical+Imaging	Imaging/Radiographic; vars 3	logistic regression	External	0.65
Maceroli et al., 2017	USA	Retrospective cohort; Single-center	Dept of Orthopedics, General hospital inpatients; Single level 1 trauma center trauma registry (2007–2016)	Lower limb; Tibia fracture nonunions and segmental bone defects (diaphyseal, post-traumatic); baseline: Established nonunion/defect	Unclear/Mixed	203	Treatment/Management outcome	> 6-12mo	Surgery event	Imaging/Radiographic; vars 5	logistic regression	Apparent	0.77
Zura et al., 2017	USA	Database study; Multi-center	US nationwide commercial/Medicare/Medicaid claims database (Truven Health Analytics); US Truven Health Analytics administrative claims database (fractures in 2011 with 12-month follow-up)	Lower limb; Upper limb; Rib; Other; Metatarsal fractures (all-cause adult metatarsal fractures); Radius fractures (all-cause adult radius fractures); Ankle fractures (adult ankle region fractures); Metacarpal fractures (adult hand fractures); Trunk fractures (rib, sternum, larynx and trachea); Tarsal fractures (midfoot/hindfoot); Humerus fractures; Tibial fractures; Radius and ulna fractures; Ulna fractures; Clavicle fractures; Scaphoid fractures; Patella fractures; Pelvic fractures; Fibula fractures; Femoral neck fractures; Combined tibia and fibula fractures; Femoral fractures (excluding neck); baseline: Acute/stress fracture	Unclear/Mixed	5022–58,377	Nonunion	> 6-12mo	Claims	Imaging/Radiographic; Clinical/Demographic; vars 5–13	logistic regression	Internal	0.62–0.73
Massari et al., 2013	Italy	Retrospective cohort; Multi-center	Dept of Orthopedics, General hospital inpatients; Multicenter hospital records (2007–2009)	Lower limb; Leg fractures (tibia, fibula, or tibia + fibula; non-osteoporotic; treated conservatively or surgically); baseline: Acute/stress fracture	Nonoperative	53	Delayed union	> 6-12mo	Clinical+Imaging	Omics/miRNA; vars 12–18	scoring system; risk score (ARRCO)	Apparent	0.62–0.82
Ramamurthy et al., 2007	UK	Retrospective cohort; Multi-center	Dept of Orthopedics, General hospital inpatients; Two orthopaedic hospital records (Apr 1991–Feb 2003; Apr 1996–Nov 2002)	Upper limb; Scaphoid nonunions treated with non-vascular bone grafting and internal fixation; baseline: Established nonunion/defect	Surgical	126	Treatment/Management outcome	> 24mo	CT/CBCT	Clinical/Demographic; vars 2	logistic regression	Apparent	0.82
Goudie et al., 2021	UK	Retrospective cohort; Single-center	Dept of Orthopedics, General hospital inpatients; Single trauma center records (development: 2002–2007; external validation: 2014–2015)	Upper limb; Proximal humeral neck fractures (isolated, nonpathologic; nonoperatively treated); baseline: Acute/stress fracture	Nonoperative	1835	Nonunion	> 3-6mo	Clinical+Imaging	Clinical/Demographic; vars 3	logistic regression	External	0.95
Santolini et al., 2020	Multi-country (UK, Italy)	Case-control; Multi-center	Dept of Orthopedics, General hospital inpatients; Two hospital orthopaedic trauma registries (Jun 2009–Sep 2016)	Lower limb; Femoral and tibial shaft fractures treated surgically with intramedullary nail, plate, or circular external fixator; baseline: Acute/stress fracture	Surgical	200	Nonunion	> 6-12mo	Clinical+Imaging	Omics/miRNA; vars 8	logistic regression	Apparent	0.92–0.94
Mundi et al., 2020	Multi-country (Canada, USA, Netherlands, Australia, India, Norway)	Retrospective cohort; Multi-center	Dept of Orthopedics, General hospital inpatients; SPRINT and FLOW randomized trial datasets	Lower limb; Tibial shaft fractures treated with intramedullary nail fixation (open and closed, post-traumatic); baseline: Acute/stress fracture	Surgical	155	Nonunion	> 6-12mo	Clinical+Imaging	Imaging/Radiographic; vars 3–4	logistic regression	Apparent	0.70–0.81
Chen et al., 2025	China	Case-control; Single-center	Dept of Orthopedics, General hospital inpatients; Single hospital records (Jan 2010–Jun 2023)	Other; Defect-type bone nonunion of long bones in the extremities (post-traumatic/post-infectious/other causes); baseline: Established nonunion/defect	Unclear/Mixed	565	Poor/non-healing	> 6-12mo	Radiograph	Imaging/Radiographic; vars 10	logistic regression	Internal	0.83
Cui et al., 2022	China	Retrospective cohort; Single-center	Dept of Orthopedics, General hospital inpatients; Single hospital records (Oct 2016–Oct 2020)	Lower limb; Hip fractures in elderly patients (femoral neck and intertrochanteric fractures; open and closed); baseline: Acute/stress fracture	Unclear/Mixed	118	Delayed union	<=3mo	Clinical+Imaging	Serum/Blood biomarkers; vars 5	logistic regression	Apparent	0.94
Guo et al., 2025	China	Retrospective cohort; Single-center	Dept of Orthopedics, General hospital inpatients; Single hospital records (Oct 2021–Apr 2024)	Lower limb; Femoral neck fractures (traumatic; open and closed); baseline: Acute/stress fracture	Unclear/Mixed	371	Delayed union	> 3-6mo	CT/CBCT	Omics/miRNA; vars 3	logistic regression	Apparent	0.93
Hao et al., 2023	China	Retrospective cohort; Multi-center	Dept of Orthopedics, General hospital inpatients; Single hospital records (Jan 2014–Dec 2020); external validation cohort from another tertiary hospital (Jan 2021–May 2022)	Lower limb; Femoral shaft fractures (adult traumatic shaft fractures treated surgically with internal fixation); baseline: Acute/stress fracture	Surgical	564	Nonunion	> 6-12mo	Clinical+Imaging	Clinical/Demographic; Omics/miRNA; vars 4	logistic regression	External	0.80
Liao et al., 2022	China	Retrospective cohort; Single-center	Dept of Orthopedics, General hospital inpatients; Single hospital records (Jan 2019–Jan 2020)	Lower limb; Traumatic tibial plateau fractures treated with posterior knee approach plate fixation; baseline: Acute/stress fracture	Surgical	80	Delayed union	> 6-12mo	Radiograph	Serum/Blood biomarkers; vars 2	logistic regression	Apparent	0.90
Liu et al., 2022	China	Retrospective cohort; Single-center	Dept of Orthopedics, General hospital inpatients; Single hospital records (May 2017–Apr 2022)	Lower limb; Knee fractures (tibial plateau, patella, distal femur) after sports-related knee injury in young gymnasts; baseline: Acute/stress fracture	Surgical	67	Nonunion	<=3mo	Clinical+Imaging	Serum/Blood biomarkers; vars 6	logistic regression	Apparent	0.91
Peng et al., 2022	China	Case-control; Single-center	Dept of Orthopedics, General hospital inpatients; Single hospital records (Jan 2020–Dec 2020)	Lower limb; Closed comminuted femoral shaft fractures (surgically treated); baseline: Acute/stress fracture	Surgical	223	Delayed union	<=3mo	CT/CBCT	Omics/miRNA; vars 3	combined biomarker ROC model (exact method NR)	Apparent	0.95
WANG et al., 2025	China	Prospective cohort; Single-center	Dept of Orthopedics, General hospital inpatients; Single hospital records (Mar 2021–Feb 2024)	Lower limb; Tibial fractures (shaft, plateau, distal; open and closed) treated surgically; baseline: Acute/stress fracture	Surgical	117	Delayed union	> 3-6mo	CT/CBCT	Serum/Blood biomarkers; vars 2	logistic regression	Apparent	0.90
ZHU et al., 2025	China	Retrospective cohort; Single-center	Dept of Orthopedics, General hospital inpatients; Single hospital records (Oct 2020—Oct 2023)	Other; Traumatic fractures (lower limb and spinal fractures; open and closed, treated with internal fixation); baseline: Acute/stress fracture	Surgical	174	Delayed union	<=3mo	Radiograph	Serum/Blood biomarkers; vars 2	logistic regression	Apparent	0.93

Abbreviations: *AUC* Area under the receiver operating characteristic curve, *AO/OTA* Arbeitsgemeinschaft fur Osteosynthesefragen/Orthopaedic Trauma Association, *CT/CBCT* computed tomography/cone-beam computed tomography, *DHS* Dynamic hip screw, *MRI* Magnetic resonance imaging, *NR* Not reported, *OVCF* Osteoporotic vertebral compression fracture, *PFNA* Proximal femoral nail anti-rotation, *PKP* Percutaneous kyphoplasty, *PVP* Percutaneous vertebroplasty, *RCT* Randomized controlled trial

A total of 97 eligible model entries were identified (Supplementary Table S8). Seventeen studies contributed more than one model [29, 30, 40, 53, 57, 78, 83–85, 90, 91, 95, 96, 99, 101–103], and one study contributed 18 models [91]. The target outcomes were nonunion (*n* = 59) and delayed union (*n* = 38). The models most frequently focused on lower-limb fractures (*n* = 54). Prediction horizons and outcome ascertainment methods varied; outcome ascertainment most often combined clinical assessment with imaging (*n* = 50), whereas 20 models relied on administrative definitions from claims or registries.

**Fig. 2:**
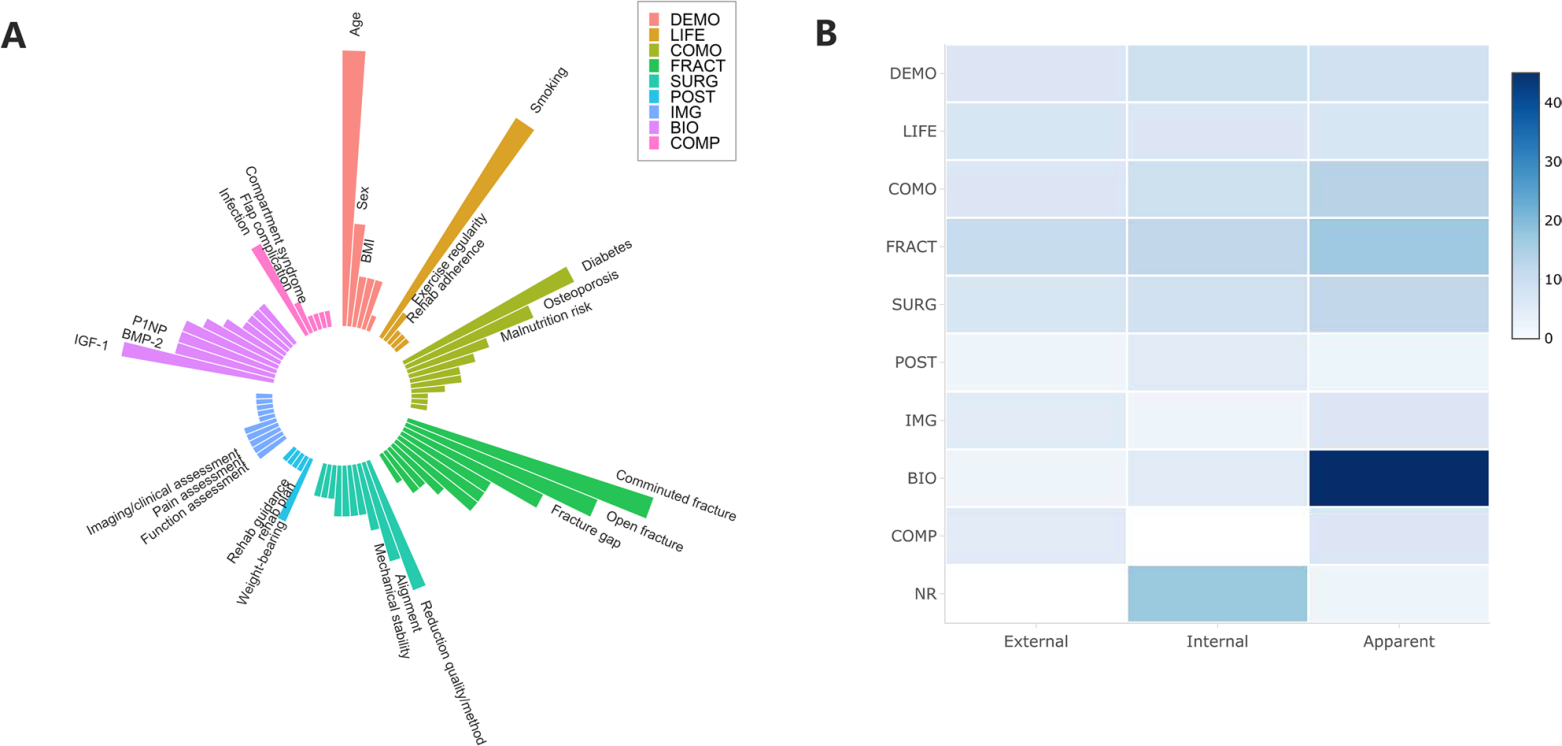
Predictor domains and their use across model performance tiers. **A** Circular bar plot summarizing the distribution of commonly used predictors across different predictor domains. **B** Heatmap showing predictor-domain coverage across model performance tiers, including apparent performance, internal validation, and external validation. DEMO, demographic factors; LIFE, lifestyle factors; COMO, comorbidities; FRACT, fracture-related factors; SURG, surgical factors; POST, postoperative factors; IMG, imaging assessment; BIO, biomarkers; COMP, complications; NR, not reported

**Fig. 3:**
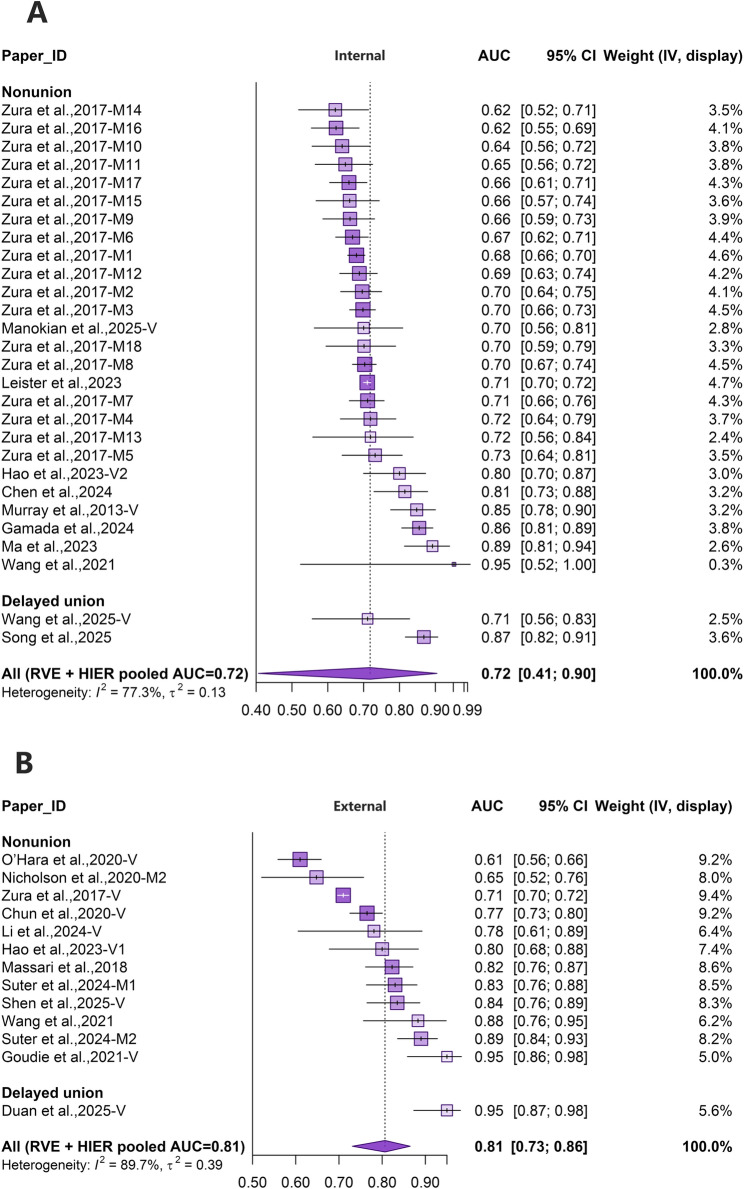
**A** Forest plots of pooled AUC for validated models: internally validated models. **B** Forest plots of pooled AUC for validated models: externally validated models

Traditional regression approaches were dominant, with logistic-regression modeling used in most models. Across the performance analyses, 97 models were synthesized and stratified as apparent performance (k = 56), internal validation (k = 28), and external validation (k = 13) (Figs. 3A and 3B). Predictors most often captured demographic and clinical characteristics, fracture parameters, and imaging-based assessments, whereas serum biomarkers were largely confined to the apparent-performance tier (Figs. 2A and 2B). The circular predictor heatmap summarizes the distribution of commonly used predictors across domains, and the domain-by-validation heatmap illustrates differences in domain coverage across validation tiers.

### Study assessment

PROBAST assessment results are presented in Table 2. Overall, all 77 included studies were at high risk of bias. Retrospective cohort designs predominated (62/77; Table 1), which may be more prone to misclassification in outcome adjudication and predictor assessment. Across domains, the analysis domain was the principal driver of bias (76/77 rated high), mainly reflecting inadequate control for overfitting and limited analytic rigor. Predictor selection was commonly based on prespecified sets and conventional screening or stepwise approaches, whereas penalized methods were rarely used (Supplementary Table S8). Validation was frequently insufficient: across all extracted entries (*n* = 97), 56/97 reported apparent performance only, 28/97 conducted internal validation, and only 13/97 reported external validation.

**Table 2:** Risk of bias and applicability of included studies (PROBAST)

Study_ID	Study	ROB: Participants	ROB: Predictors	ROB: Outcome	ROB: Analysis	ROB: Overall	App: Participants	App: Predictors	App: Outcome	App: Overall
2	Shen et al.,2025	+	+	?	−	−	+	+	+	+
3	Chen et al.,2024	+	+	+	−	−	+	+	+	+
4	Chun et al.,2020	+	+	+	−	−	+	+	+	+
5	Deng et al.,2019	+	+	+	−	−	+	+	+	+
7	Guo et al.,2024	+	+	+	−	−	+	+	+	+
8	Guo et al.,2023	+	+	+	−	−	+	+	+	+
9	Guo et al.,2024	+	+	+	−	−	+	+	+	+
10	Hao et al.,2024	+	+	+	−	−	+	+	+	+
11	He et al.,2024	+	+	+	−	−	+	+	+	+
12	Huo et al.,2024	+	+	+	−	−	+	+	+	+
14	Jiang et al.,2025	+	+	+	−	−	+	+	+	+
15	Jin et al.,2025	+	+	+	−	−	+	+	+	+
17	Liang et al.,2024	+	+	+	−	−	+	+	+	+
18	Liao et al.,2024	+	+	+	−	−	+	+	+	+
19	Liu et al.,2025	+	+	+	−	−	+	+	+	+
20	Liu et al.,2023	+	+	+	−	−	+	+	+	+
21	Liu et al.,2025	+	+	+	−	−	+	+	+	+
23	Ma et al.,2023	+	+	+	−	−	+	+	+	+
24	Qian et al.,2022	+	+	+	−	−	+	+	+	+
25	Ren et al.,2022	+	+	+	−	−	+	+	+	+
26	Su et al.,2022	−	?	?	−	−	+	+	+	+
27	Wang et al.,2022	−	?	?	−	−	+	+	+	+
28	Wang et al.,2024	+	+	+	−	−	+	+	+	+
29	Wang et al.,2021	+	+	+	−	−	+	+	+	+
30	Wang et al.,2024	−	+	?	−	−	+	+	+	+
33	Xu et al.,2021	+	+	+	−	−	+	+	+	+
35	Yang et al.,2020	+	+	+	−	−	+	+	+	+
36	Yang et al.,2024	+	+	+	−	−	+	+	+	+
37	Ye et al.,2023	+	+	+	−	−	+	+	+	+
39	Yuan et al.,2024	−	?	+	−	−	+	+	+	+
41	Zhang et al.,2023	+	+	+	−	−	+	+	+	+
43	Zhang et al.,2022	+	+	+	−	−	+	+	+	+
45	Zhang et al.,2024	+	+	+	−	−	+	+	+	+
47	Zhao et al.,2023	−	+	?	−	−	+	+	+	+
48	Zheng et al.,2024	+	+	+	−	−	+	+	+	+
51	Zhou et al.,2024	+	+	+	−	−	+	+	+	+
54	Wang et al.,2025	+	+	+	−	−	+	+	+	+
55	Manokian et al.,2025	+	+	+	−	−	+	+	+	+
56	Tang et al.,2025	+	+	+	−	−	+	+	+	+
60	Braun et al.,2025	+	+	+	−	−	+	+	+	+
61	Gamada et al.,2024	?	+	+	−	−	+	+	+	+
63	Song et al.,2025	+	+	+	−	−	+	+	+	+
64	Zhu et al.,2025	+	+	+	−	−	+	+	+	+
65	Wu et al.,2025	+	+	+	−	−	+	+	+	+
66	Xiao et al.,2024	+	+	+	−	−	+	+	+	+
71	Leister et al.,2023	−	+	+	−	−	+	+	+	+
75	Han et al.,2025	−	+	+	−	−	+	+	+	+
76	Hsu et al.,2016	+	+	+	−	−	+	+	+	+
77	Haubruck et al.,2018	−	−	+	−	−	+	+	+	+
79	Kraus et al.,2024	−	+	+	−	−	+	+	+	+
80	Li et al.,2024	−	+	+	−	−	+	+	+	+
81	Suter et al.,2024	−	+	+	−	−	+	+	+	+
82	Panteli et al.,2021	+	+	+	−	−	+	+	+	+
83	Duan et al.,2025	−	+	+	−	−	+	+	+	+
84	Wang et al.,2021	+	+	+	−	−	+	+	+	+
86	Zura et al.,2017	+	?	−	−	−	+	?	?	?
87	Murray et al.,2013	+	+	+	−	−	+	+	+	+
89	Zhu et al.,2021	−	+	+	−	−	+	+	−	−
90	Massari et al.,2018	+	+	−	+	−	+	+	+	+
91	O’Hara et al.,2020	+	+	+	−	−	+	+	+	+
92	Nicholson et al.,2020	+	+	−	−	−	+	+	+	+
94	Maceroli et al.,2017	−	+	+	−	−	+	+	?	?
96	Zura et al.,2017	+	+	−	−	−	+	+	+	+
97	Massari et al.,2013	−	+	?	−	−	+	+	+	+
99	Ramamurthy et al.,2007	+	+	+	−	−	+	+	+	+
100	Goudie et al.,2021	+	+	+	−	−	+	+	+	+
102	Santolini et al.,2020	?	+	+	−	−	+	+	+	+
104	Mundi et al.,2020	−	+	+	−	−	−	+	+	−
105	Chen et al.,2025	+	+	+	−	−	+	+	+	+
106	Cui et al.,2022	?	+	?	−	−	+	+	?	−
108	Guo et al.,2025	+	+	?	−	−	+	+	+	+
109	Hao et al.,2023	+	+	+	−	−	+	+	+	+
111	Liao et al.,2022	+	+	?	−	−	+	+	+	+
112	Liu et al.,2022	+	+	+	−	−	+	+	+	+
113	Peng et al.,2022	−	+	+	−	−	+	+	+	+
114	Wang et al.,2025	+	+	+	−	−	+	+	+	+
116	Zhu et al.,2025	?	+	+	−	−	+	+	+	+

Abbreviations: *PROBAST* Prediction model Risk Of Bias ASsessment Tool, *ROB* Risk of bias, *App* Applicability

Symbols: + low risk of bias/low concern regarding applicability, − high risk of bias/high concern regarding applicability, ? unclear risk of bias/unclear concern regarding applicability

Calibration was infrequently reported and was typically limited to calibration plots or Hosmer–Lemeshow testing; 25/97 models reported any calibration assessment in development, and among models with internal or external validation (41 models), 10/41 reported calibration on the validation data. Missing data handling was seldom described (78/97 not reported); when reported, complete-case analysis was most common (17/97) and multiple imputation was rare (2/97). Limited events per variable were also frequent (EPV < 10 in 20/92 models with calculable EPV), raising concerns about optimistic performance estimates. In contrast, applicability concerns were generally low (72/77 rated low overall; Table 2).

### Meta-analysis

#### Development (apparent) performance

In the apparent-performance meta-analysis (Supplementary Figure S3), the pooled AUC was 0.88 (95% CI 0.86–0.90), with substantial heterogeneity (I² = 91.9%, τ² = 0.33). Leave-one-out sensitivity analyses indicated that omitting any single study did not materially change the pooled estimate. Funnel-plot diagnostics yielded a significant Egger’s test (*p* = 5.16 × 10⁻⁸) but a non-significant rank correlation test (*p* = 0.158).

### Validation performance

For models with internal validation (Fig. 3A), the pooled AUC was 0.72 (95% CI 0.41–0.90), with substantial heterogeneity (I² = 77.3%, τ² = 0.13). Leave-one-out analyses indicated stable pooled estimates. Funnel-plot diagnostics were mixed (Egger’s test, *p* = 0.00915; rank correlation, *p* = 0.484). For external validation (Fig. 3B), the pooled AUC was 0.81 (95% CI 0.73–0.86), with substantial heterogeneity (I² = 89.7%, τ² = 0.39). Leave-one-out analyses again suggested stable pooled estimates, and funnel-plot diagnostics showed a significant Egger’s test (*p* = 0.0319) but a non-significant rank correlation test (*p* = 0.217).

### Optimism in discrimination (development versus validation)

To assess potential optimism in model discrimination, we extracted paired AUCs from studies reporting both development (training) AUC and validated AUC (internal or external) and calculated optimism as AUC_train − AUC_validated. Twelve models from 11 studies reported both training and validated AUCs, yielding 12 paired comparisons in total (8 external and 4 internal validations) (Supplementary Table S8). Overall, training AUCs were generally higher than validated AUCs, with a mean optimism of 0.052 (SD 0.075), a median of 0.023 (IQR 0.014–0.041), and values ranging from − 0.014 to 0.240. When stratified by validation type, mean optimism was 0.062 for external validation and 0.030 for internal validation. The paired scatterplot of training versus validated AUCs is provided in Supplementary Figure S2.

### Anatomical subgroup summary

Table 3 presents model performance by anatomical subgroup and validation tier. The included model entries covered a broad range of fracture settings, from appendicular shaft and proximal femoral fractures to axial skeletal injuries and multisite cohorts. Because several subgroups were small, especially in the external-validation tier, the results are reported descriptively.

**Table 3:** Descriptive subgroup summary of model discrimination by anatomical subgroup and validation tier

Validation tier	Anatomical subgroup	Models / studies	AUC, median [IQR]	Calibration reported
Apparent	Appendicular shaft / diaphyseal long-bone	20 / 20	0.84 [0.80–0.91]	9/20
Apparent	Multisite / broad anatomical mix	9 / 9	0.93 [0.86–0.94]	1/9
Apparent	Unclear / insufficiently specific segment	8 / 8	0.89 [0.87–0.90]	0/8
Apparent	Other site-specific appendicular	7 / 7	0.90 [0.87–0.92]	2/7
Apparent	Proximal femur / hip	7 / 7	0.86 [0.83–0.92]	1/7
Apparent	Axial skeleton	5 / 5	0.88 [0.86–0.88]	0/5
Internal	Other site-specific appendicular	12 / 12	0.68 [0.66–0.70]	1/12
Internal	Appendicular shaft / diaphyseal long-bone	6 / 6	0.75 [0.70–0.83]	3/6
Internal	Axial skeleton	4 / 4	0.79 [0.73–0.86]	0/4
Internal	Unclear / insufficiently specific segment	4 / 4	0.70 [0.69–0.70]	0/4
Internal	Proximal femur / hip	2 / 2	0.72 [0.67–0.77]	0/2
External	Appendicular shaft / diaphyseal long-bone	7 / 7	0.78 [0.71–0.81]	4/7
External	Multisite / broad anatomical mix	3 / 3	0.83 [0.77–0.89]	1/3
External	Other site-specific appendicular	1 / 1	0.95 [0.95–0.95]	0/1
External	Proximal femur / hip	1 / 1	0.88 [0.88–0.88]	1/1
External	Unclear / insufficiently specific segment	1 / 1	0.82 [0.82–0.82]	0/1

“Models / studies” indicates the number of eligible model entries / contributing studies; “Calibration reported” indicates the number of model entries reporting any calibration assessment / total model entries within that subgroup

Abbreviations: *AUC* Area under the receiver operating characteristic curve, *IQR* Interquartile range

### Meta-regression

To explore potential drivers of between-study heterogeneity in discrimination for nonunion models, we conducted a multilevel meta-regression of logit-transformed AUC estimates. The model used a random-effects structure to account for clustering of multiple models within studies, with robust clustered inference at the study level. Given that external validation was uncommon across the evidence base, validation tier was included as a moderator to account for systematic differences in performance estimates across validation designs when synthesizing across studies. In the nonunion meta-regression dataset (k = 59 performance estimates), we assessed whether discrimination varied systematically by validation tier (external vs. internal vs. apparent), outcome-horizon group (≤ 3 months, > 3–6 months, and > 6–12 months), inclusion of biological-domain predictors, and sample size (log-transformed).

**Fig. 4:**
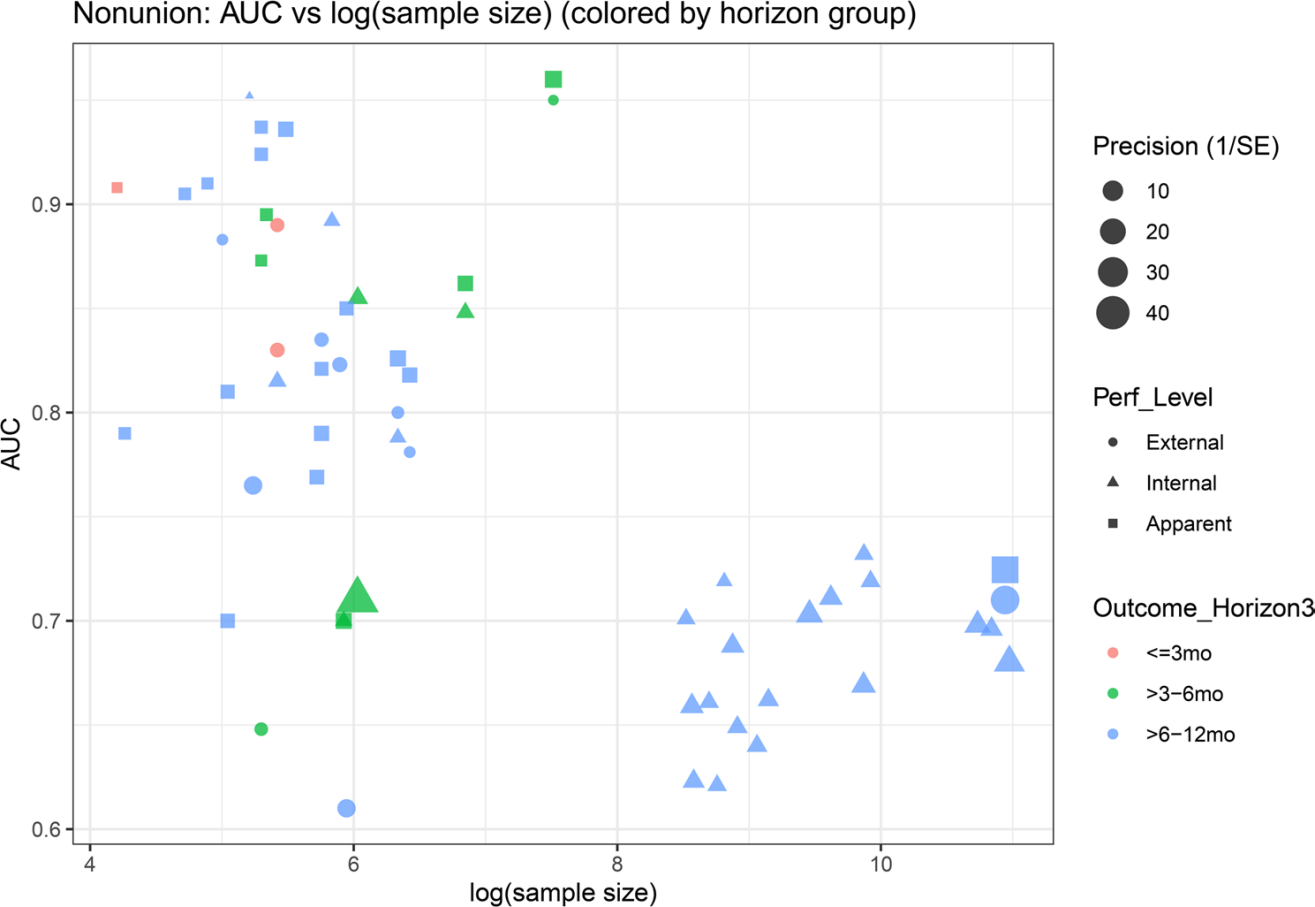
Association between AUC and sample size, stratified by performance tier

Overall, validation tier and predictor-domain composition explained part of the observed heterogeneity. Apparent-performance estimates tended to be higher than externally validated estimates, whereas internal validation did not show a clear difference from external validation after adjustment for other covariates. Models incorporating biological-domain predictors showed higher discrimination on average. In contrast, neither outcome-horizon group nor sample size demonstrated a consistent independent association with AUC, suggesting that heterogeneity was not explained by follow-up window or study scale alone once validation tier and predictor domain were considered. Figure 4 provides a complementary visual summary of these patterns. The scatter of AUC across sample sizes is wide, but higher AUC values are more frequently observed among apparent-performance estimates, consistent with more optimistic estimates when evaluated in the development data. By comparison, estimates from validation-based tiers cluster at more moderate AUC levels. Notably, large-sample studies do not uniformly exhibit higher discrimination, underscoring that study scale should not be interpreted as a proxy for model performance without considering validation design and predictor set composition.

### Certainty of evidence (GRADE)

Based on GRADE, the certainty of evidence for discrimination (AUC) was very low across apparent, internally validated, and externally validated performance estimates. This was driven by very serious risk of bias based on PROBAST. In particular, concerns were concentrated in the analysis domain, and reporting of calibration and missing-data handling was often incomplete. Additional downgrading reflected substantial between-study heterogeneity, marked imprecision for internally validated estimates, and signals of small-study effects.

## Discussion

This systematic review and meta-analysis aimed to identify and synthesize the current evidence on prediction models developed to forecast nonunion or delayed union. We included 77 studies and extracted 97 eligible prediction models, which we grouped by evidence tier into apparent performance only (*n* = 56), internal validation (*n* = 28), and external validation (*n* = 13). Overall, discrimination appeared strong at the development stage: across 56 development models, the pooled AUC was 0.88 (95% CI 0.86–0.90), although heterogeneity was substantial (I²=91.9%). In contrast, performance generally attenuated under validation. The pooled AUC was 0.81 for externally validated models (95% CI 0.73–0.86; I²=89.7%) and 0.72 for internally validated models (95% CI 0.41–0.90; I²=77.3%). These pooled AUCs, however, should not be interpreted as transportable benchmarks of clinical performance, given the scarcity of independent external validation [29, 30, 40, 53, 63, 78, 83, 85, 90, 94, 99, 102], the strong between-study heterogeneity, and the overall high risk of bias indicated by PROBAST (Table 2). Discrimination alone cannot establish whether predicted probabilities are sufficiently calibrated or clinically useful. Overall, the current evidence is more consistent with a detectable predictive signal in a broad and conceptually heterogeneous body of literature than with a directly deployable and reliable tool.

Consistent with patterns widely observed in the prediction model literature, we also found that discrimination in development datasets was often higher than in validation datasets. Importantly, this gap was not merely random fluctuation. Among 12 paired development and validation AUC comparisons extracted for the same model, the development AUC exceeded the validation AUC by a mean of 0.052 (SD 0.075), indicating systematic optimism at development. In some studies, the drop from development to validation exceeded 0.15 [29, 102], suggesting that model performance was highly sensitive to cohort differences and evaluation conditions. Several overlapping factors likely contribute. Many models considered relatively large candidate predictor sets despite limited event counts, a setting that can destabilize coefficient estimates and inflate apparent AUC. Predictor construction also frequently relied on selection-driven strategies such as univariable screening or stepwise regression, which are prone to overoptimistic development performance and limited reproducibility. In addition, internal validation commonly used split-sample designs, which reduce the effective development sample and increase the variability of validated estimates when total sample size is modest. These patterns suggest that the more impressive development AUCs likely reflect structural optimism in the evidence-generation pathway, whereas the attenuation under validation may more closely approximate reproducible performance.

From the perspective of methodological quality, this pattern aligns closely with structural weaknesses in the analytic foundations of the evidence base. Based on PROBAST, the included studies generally carried high risk of bias, driven primarily by the analysis domain, indicating that many performance estimates were generated within analytic frameworks that were insufficiently robust and difficult to reproduce. Missing-data handling was not adequately reported in most studies, which may increase sensitivity to sample composition and undermine generalizability. Calibration assessment was also sparse in both development and validation, and when reported it was often limited to Hosmer–Lemeshow testing or coarse calibration plots. Without calibration intercepts and slopes, even models with acceptable discrimination cannot be judged on whether predicted probabilities are reliable enough to support threshold-based decisions. Finally, external validation was rare, limiting assessment of transportability across populations, centers, and time. For these reasons, development discrimination should not be interpreted as evidence of broadly generalizable clinical utility.

Beyond analytical optimism, heterogeneity in this review also reflects limited comparability in outcomes, fracture settings, and treatment contexts. Nonunion and delayed union are related but clinically distinct endpoints, and their definitions varied across studies in both time threshold and ascertainment. Outcome horizons ranged from ≤ 3 months to > 24 months, and ascertainment methods spanned combined clinical and imaging assessment, imaging-based assessment, and administrative proxies. The included models also covered markedly different fracture settings, including appendicular shaft fractures, proximal femoral fractures, multisite cohorts, and axial skeletal injuries such as vertebral fractures, spondylolysis, and odontoid fractures. These entities differ in loading environment, healing biology, and treatment pathway, which limits direct comparability. Transportability is further constrained by variation in case-mix, treatment strategy, and study setting, as most studies were retrospective, single-center, and conducted in China. Because healthcare systems, fracture populations, treatment pathways, follow-up practices, and implementation settings differ internationally, this geographical concentration may further limit generalizability outside the contexts in which most models were developed. Consequently, these differences mean that pooled discrimination estimates should be interpreted cautiously and not as interchangeable across distinct fracture settings.

In this context, the central contribution of our study is not to select a single highest-performing model for immediate clinical implementation, but to clarify when prediction models for fracture healing might truly deliver clinical value and which evidence components remain missing for clinical readiness. For clinicians, the current evidence should be viewed as a signal of potential and a basis for further validation research, not as support for adopting existing models as ready-to-use decision-support tools. From a decision-making perspective, prediction models are most meaningful within a modifiable management window, namely, identifying high-risk individuals before nonunion becomes established, in order to guide intensified follow-up and imaging, optimization of mechanical stability and modifiable risk factors, and, when appropriate, earlier referral or planned intervention. In this setting, model value should not be judged by discrimination alone, but also by whether predicted probabilities are well calibrated and whether the model yields net benefit across clinically plausible thresholds. Future work should therefore prioritize clearer outcome definitions and assessment windows, independent external validation with routine calibration reporting and recalibration when needed, stronger control of missing data and overfitting during development, and evaluation of clinical utility on external data.

### Limitations

This review has several limitations. External validation remained limited, and internal validation often relied on split-sample designs, which can yield unstable estimates when effective sample size is reduced. Outcome definitions and assessment conditions were not fully comparable across studies, with wide variation in time horizons and ascertainment methods. We were also unable to stratify models more finely by loading environment or fracture-healing biology, because these features were incompletely and inconsistently reported in the primary studies. Likewise, classification of delayed union and nonunion necessarily relied on the definitions used in the original reports. The included models also varied in how prediction constructs were specified and reported, which further limited conceptual comparability. Missing-data handling and calibration on validation data were often insufficiently reported, further weakening confidence in threshold-based use. Accordingly, the pooled AUCs are best interpreted as signals of potential rather than evidence of clinical readiness.

## Conclusion

This systematic review and meta-analysis summarizes the current evidence on prediction models for nonunion and delayed union. Overall, models often perform well in development but show attenuated and less certain discrimination under stricter validation. Limited external validation, clinical and anatomical heterogeneity, inconsistent outcome definitions and ascertainment, a high prevalence of analysis-domain bias, and sparse reporting of missing-data handling and calibration mean that pooled AUCs should be viewed as signals of potential rather than evidence of clinical readiness. Future work should prioritize clearer and more harmonized outcome definitions and time windows, routine external validation with calibration reporting, and evaluation of clinical utility before such models can support deployable decision-support tools.

## Supplementary Information

Supplementary Material 1.

Supplementary Material 2.

## Data Availability

All data generated or analysed during this study are included in this published article and its supplementary information files.
