# Supplementary material for: Performance of prediction models for delayed union and nonunion after fracture: a systematic review and meta-analysis
Source: BMC Musculoskelet Disord. 2026 May 29;27:487. doi: 10.1186/s12891-026-09941-4 (PMC13242139; doi:10.1186/s12891-026-09941-4)
Supplement: Supplementary file 1 — Supplementary Material 1. [file 12891_2026_9941_MOESM1_ESM.docx]

| **Section and Topic** | **Item #** | **Checklist item** | **Location where item is reported** |
| --- | --- | --- | --- |
| **TITLE** | | |  |
| Title | 1 | Identify the report as a systematic review. | Title (Page 1) |
| **ABSTRACT** | | |  |
| Abstract | 2 | See the PRISMA 2020 for Abstracts checklist. | Abstract (Pages 1-2) |
| **INTRODUCTION** | | |  |
| Rationale | 3 | Describe the rationale for the review in the context of existing knowledge. | Introduction (Pages 3-5) |
| Objectives | 4 | Provide an explicit statement of the objective(s) or question(s) the review addresses. | Abstract (Page 1); Introduction (Page 5) |
| **METHODS** | | |  |
| Eligibility criteria | 5 | Specify the inclusion and exclusion criteria for the review and how studies were grouped for the syntheses. | Methods - Eligibility criteria (Pages 6-7) |
| Information sources | 6 | Specify all databases, registers, websites, organisations, reference lists and other sources searched or consulted to identify studies. Specify the date when each source was last searched or consulted. | Methods - Search strategy (Page 6) |
| Search strategy | 7 | Present the full search strategies for all databases, registers and websites, including any filters and limits used. | Supplementary File (Search strategies) |
| Selection process | 8 | Specify the methods used to decide whether a study met the inclusion criteria of the review, including how many reviewers screened each record and each report retrieved, whether they worked independently, and if applicable, details of automation tools used in the process. | Methods - Study selection and data collection process (Page 7) |
| Data collection process | 9 | Specify the methods used to collect data from reports, including how many reviewers collected data from each report, whether they worked independently, any processes for obtaining or confirming data from study investigators, and if applicable, details of automation tools used in the process. | Methods - Study selection and data collection process (Page 7) |
| Data items | 10a | List and define all outcomes for which data were sought. Specify whether all results that were compatible with each outcome domain in each study were sought (e.g. for all measures, time points, analyses), and if not, the methods used to decide which results to collect. | Methods - Study selection and data collection process (Page 7); Methods - Statistical analyses (Pages 8-9) |
|  | 10b | List and define all other variables for which data were sought (e.g. participant and intervention characteristics, funding sources). Describe any assumptions made about any missing or unclear information. | Methods - Study selection and data collection process (Page 7) |
| Study risk of bias assessment | 11 | Specify the methods used to assess risk of bias in the included studies, including details of the tool(s) used, how many reviewers assessed each study and whether they worked independently, and if applicable, details of automation tools used in the process. | Methods - Quality assessment and certainty of evidence (Pages 7-8) |
| Effect measures | 12 | Specify for each outcome the effect measure(s) (e.g. risk ratio, mean difference) used in the synthesis or presentation of results. | Methods - Statistical analyses (Pages 8-9) |
| Synthesis methods | 13a | Describe the processes used to decide which studies were eligible for each synthesis (e.g. tabulating the study intervention characteristics and comparing against the planned groups for each synthesis (item #5)). | Results - Study characteristics (Pages 10-11); Results - Meta-analysis (Pages 12-14) |
|  | 13b | Describe any methods required to prepare the data for presentation or synthesis, such as handling of missing summary statistics, or data conversions. | Methods - Study selection and data collection process (Page 7); Methods - Statistical analyses (Pages 8-9) |
|  | 13c | Describe any methods used to tabulate or visually display results of individual studies and syntheses. | Methods - Statistical analyses (Page 8); Results (Pages 10-15); Figure legends (Page 33); Tables 1-2 (Pages 34-59) |
|  | 13d | Describe any methods used to synthesize results and provide a rationale for the choice(s). If meta-analysis was performed, describe the model(s), method(s) to identify the presence and extent of statistical heterogeneity, and software package(s) used. | Methods - Statistical analyses (Pages 8-9) |
|  | 13e | Describe any methods used to explore possible causes of heterogeneity among study results (e.g. subgroup analysis, meta-regression). | Methods - Statistical analyses (Page 9); Results - Meta-regression (Pages 13-14) |
|  | 13f | Describe any sensitivity analyses conducted to assess robustness of the synthesized results. | Results - Meta-analysis (Pages 12-14); Supplementary materials (sensitivity analyses as cited) |
| Reporting bias assessment | 14 | Describe any methods used to assess risk of bias due to missing results in a synthesis (arising from reporting biases). | Methods - Statistical analyses (Page 9); Results - Meta-analysis (Pages 12-14) |
| Certainty assessment | 15 | Describe any methods used to assess certainty (or confidence) in the body of evidence for an outcome. | Methods - Quality assessment and certainty of evidence (Pages 7-8) |
| **RESULTS** | | |  |
| Study selection | 16a | Describe the results of the search and selection process, from the number of records identified in the search to the number of studies included in the review, ideally using a flow diagram. | Results - Study selection (Page 10); Figure 1 legend (Page 33) |
|  | 16b | Cite studies that might appear to meet the inclusion criteria, but which were excluded, and explain why they were excluded. | Results - Study selection (Page 10) (full-text exclusions summarized; individual excluded-study list not provided) |
| Study characteristics | 17 | Cite each included study and present its characteristics. | Results - Study characteristics (Pages 10-11); Table 1 (Pages 34-55) |
| Risk of bias in studies | 18 | Present assessments of risk of bias for each included study. | Results - Study assessment (Pages 11-12); Table 2 (Pages 56-59) |
| Results of individual studies | 19 | For all outcomes, present, for each study: (a) summary statistics for each group (where appropriate) and (b) an effect estimate and its precision (e.g. confidence/credible interval), ideally using structured tables or plots. | Results - Meta-analysis (Pages 12-14); Figure legends (Page 33); Supplementary Tables/Figures as cited |
| Results of syntheses | 20a | For each synthesis, briefly summarise the characteristics and risk of bias among contributing studies. | Results - Meta-analysis (Pages 12-14); Results - Study characteristics (Pages 10-11); Results - Study assessment (Pages 11-12) |
|  | 20b | Present results of all statistical syntheses conducted. If meta-analysis was done, present for each the summary estimate and its precision (e.g. confidence/credible interval) and measures of statistical heterogeneity. If comparing groups, describe the direction of the effect. | Results - Meta-analysis (Pages 12-14); Figure legends (Page 33) |
|  | 20c | Present results of all investigations of possible causes of heterogeneity among study results. | Results - Meta-regression (Pages 13-14) |
|  | 20d | Present results of all sensitivity analyses conducted to assess the robustness of the synthesized results. | Results - Meta-analysis (Pages 12-14); Supplementary materials (sensitivity analyses as cited) |
| Reporting biases | 21 | Present assessments of risk of bias due to missing results (arising from reporting biases) for each synthesis assessed. | Results - Meta-analysis (Pages 12-14); Discussion (Pages 16-21) |
| Certainty of evidence | 22 | Present assessments of certainty (or confidence) in the body of evidence for each outcome assessed. | Results - Certainty of evidence (GRADE) (Page 15); Discussion (Pages 16-21); Abstract (Pages 1-2) |
| **DISCUSSION** | | |  |
| Discussion | 23a | Provide a general interpretation of the results in the context of other evidence. | Discussion (Pages 16-21) |
|  | 23b | Discuss any limitations of the evidence included in the review. | Discussion (Pages 16-21); Limitations (Page 22) |
|  | 23c | Discuss any limitations of the review processes used. | Limitations (Page 22) |
|  | 23d | Discuss implications of the results for practice, policy, and future research. | Discussion (Pages 16-21); Conclusion (Page 23) |
| **OTHER INFORMATION** | | |  |
| Registration and protocol | 24a | Provide registration information for the review, including register name and registration number, or state that the review was not registered. | Abstract (Page 1) (registration number withheld for double-anonymous peer review); Methods - Study design and registration (Page 6) |
|  | 24b | Indicate where the review protocol can be accessed, or state that a protocol was not prepared. | Methods - Study design and registration (Page 6) |
|  | 24c | Describe and explain any amendments to information provided at registration or in the protocol. | Methods - Study design and registration (Page 6) (Note: No amendments explicitly described in text) |
| Support | 25 | Describe sources of financial or non-financial support for the review, and the role of the funders or sponsors in the review. | Title Page (separate file; not in blinded manuscript) |
| Competing interests | 26 | Declare any competing interests of review authors. | Title Page (separate file; not in blinded manuscript) |
| Availability of data, code and other materials | 27 | Report which of the following are publicly available and where they can be found: template data collection forms; data extracted from included studies; data used for all analyses; analytic code; any other materials used in the review. | Title Page / Data Statement (separate files; not in blinded manuscript); Supplementary materials |

*From:*  Page MJ, McKenzie JE, Bossuyt PM, Boutron I, Hoffmann TC, Mulrow CD, et al. The PRISMA 2020 statement: an updated guideline for reporting systematic reviews. BMJ 2021;372:n71. doi: 10.1136/bmj.n71 For more information, visit: <http://www.prisma-statement.org/>
