# Supplementary material for: Performance of prediction models for delayed union and nonunion after fracture: a systematic review and meta-analysis
Source: BMC Musculoskelet Disord. 2026 May 29;27:487. doi: 10.1186/s12891-026-09941-4 (PMC13242139; doi:10.1186/s12891-026-09941-4)
Supplement: Supplementary file 2 — Supplementary Material 2. [file 12891_2026_9941_MOESM2_ESM.docx]

**Supplementary Material**

# Table of Contents

[Table of Contents 1](#_Toc222959757)

[Supplementary Figures 7](#_Toc222959758)

[Figure S1. calibration reporting rate 7](#_Toc222959759)

[Figure S2. optimism train vs validated 8](#_Toc222959760)

[Figure S3. Forest plot of pooled AUC for apparent (development) performance 9](#_Toc222959761)

[Figure S4. Leave-one-out sensitivity analyses for pooled AUC estimates 10](#_Toc222959762)

[Figure S5. Funnel plot for small-study effects in apparent (development) performance 11](#_Toc222959763)

[Figure S6. Funnel plot for small-study effects in internal validation 12](#_Toc222959764)

[Figure S7. Funnel plot for small-study effects in external validation 13](#_Toc222959765)

[Table S8. Traceability lists for included studies cited in the Results and Discussion (Author, year). 14](#_Toc222959766)

Table S2. Search strategies

S2 Table. Search strategies.

### Ovid Medline

Starting date: Database creation date

Searched on: November 30, 2025

Results: 1687

| **Search** | **Query** | **Results** |
| --- | --- | --- |
| #1 | exp Fractures, Ununited/ OR exp Pseudarthrosis/ OR exp Fracture Healing/ | 26022 |
| #2 | (nonunion* OR "non union*" OR "non-union*" OR nonunited OR "non-united" OR "delayed union*" OR "delayed fracture healing" OR "delayed bone healing" OR malunion* OR "mal-union*" OR pseudoarthros* OR pseudarthros* OR (fractur* adj3 heal*) OR "nonhealing fractur*" OR "non-healing fractur*").ti,ab,kf. | 48786 |
| #3 | ("delayed healing" AND (fractur* OR bone* OR orthop* OR osteotom* OR osteosynthes* OR arthrodes*)).ti,ab,kf. | 966 |
| #4 | #1 OR #2 OR #3 | 58808 |
| #5 | exp Models, Statistical/ or exp ROC Curve/ or exp Machine Learning/ or exp Artificial Intelligence/ | 789978 |
| #6 | ((predict* or prognos* or risk* or probabilit*) adj3 (model* or score* or scor* or index or tool* or equation* or calculator* or nomogram* or "prediction rule*" or "decision rule*" or "risk stratification") or risk model* or risk prediction or risk assessment or risk scor* or "risk score*" or "risk prediction model*" or predictive model* or prediction model* or predictive scor* or prediction tool* or prognostic model* or "prognostic index*" or "prognostic scor*" or "risk calculator*" or nomogram* or "scoring system*" or "score system*" or "clinical prediction rule*" or "clinical decision rule*" or multivaria* predict* or multivaria* model* or multivaria* prognos* or machine learning or "ML model*" or "ML-based" or "AI-based" or "AI-assisted" or "artificial intelligence" or deep learning or "neural network*" or "convolutional neural network*" or CNN or "recurrent neural network*" or RNN or "random forest*" or "decision tree*" or "gradient boosting" or XGBoost or "support vector machine*" or SVM or "k-nearest neighbor*" or "k nearest neighbor*" or "naive bayes" or "CatBoost" or "ensemble learning" or "supervised learning" or "unsupervised learning" or "boosted tree*" or "kernel machine*" or radiomic* or "radiograph* texture*" or "radiograph* feature*").ti,ab,kf. | 1107047 |
| #7 | #5 or #6 | 1640700 |
| #8 | exp Fractures, Bone/ | 224385 |
| #9 | fractur*.ti,ab,kf. | 358696 |
| #10 | #8 or #9 | 401774 |
| #11 | #4 and #7 and #10 | 1687 |

### EMBASE

Starting date: Database creation date

Searched on: November 30, 2025

Results: 2869

| **Search** | **Query** | **Results** |
| --- | --- | --- |
| #1 | 'fracture healing'/exp | 72963 |
| #2 | nonunion*:ti,ab,kw OR 'non union*':ti,ab,kw OR 'non-union*':ti,ab,kw OR nonunited:ti,ab,kw OR 'non-united':ti,ab,kw OR 'delayed union*':ti,ab,kw OR 'delayed fracture healing':ti,ab,kw OR 'delayed bone healing':ti,ab,kw OR malunion*:ti,ab,kw OR 'mal-union*':ti,ab,kw OR pseudoarthros*:ti,ab,kw OR pseudarthros*:ti,ab,kw OR 'nonhealing fractur*':ti,ab,kw OR 'non-healing fractur*':ti,ab,kw OR ((fractur* NEAR/3 heal*):ti,ab,kw) | 59705 |
| #3 | #1 OR #2 | 94144 |
| #4 | fracture'/exp | 445665 |
| #5 | fractur*:ti,ab,kw | 449584 |
| #6 | #4 OR #5 | 565760 |
| #7 | statistical model'/exp OR 'receiver operating characteristic'/exp OR 'machine learning'/exp OR 'artificial intelligence'/exp | 1715997 |
| #8 | (((predict* OR prognos* OR risk* OR probabilit*) NEAR/3 (model* OR score* OR scor* OR index OR tool* OR equation* OR calculator* OR nomogram* OR 'prediction rule*' OR 'decision rule*' OR 'risk stratification')):ti,ab,kw) OR 'risk model*':ti,ab,kw OR 'risk prediction':ti,ab,kw OR 'risk assessment':ti,ab,kw OR 'risk scor*':ti,ab,kw OR 'risk score*':ti,ab,kw OR 'risk prediction model*':ti,ab,kw OR 'predictive model*':ti,ab,kw OR 'prediction model*':ti,ab,kw OR 'predictive scor*':ti,ab,kw OR 'prediction tool*':ti,ab,kw OR 'prognostic model*':ti,ab,kw OR 'prognostic index*':ti,ab,kw OR 'prognostic scor*':ti,ab,kw OR 'risk calculator*':ti,ab,kw OR nomogram*:ti,ab,kw OR 'scoring system*':ti,ab,kw OR 'score system*':ti,ab,kw OR 'clinical prediction rule*':ti,ab,kw OR 'clinical decision rule*':ti,ab,kw OR 'multivaria* predict*':ti,ab,kw OR 'multivaria* model*':ti,ab,kw OR 'multivaria* prognos*':ti,ab,kw OR 'machine learning':ti,ab,kw OR 'ml model*':ti,ab,kw OR 'ml-based':ti,ab,kw OR 'ai-based':ti,ab,kw OR 'ai-assisted':ti,ab,kw OR 'artificial intelligence':ti,ab,kw OR 'deep learning':ti,ab,kw OR 'neural network*':ti,ab,kw OR 'convolutional neural network*':ti,ab,kw OR cnn:ti,ab,kw OR 'recurrent neural network*':ti,ab,kw OR rnn:ti,ab,kw OR 'random forest*':ti,ab,kw OR 'decision tree*':ti,ab,kw OR 'gradient boosting':ti,ab,kw OR xgboost:ti,ab,kw OR 'support vector machine*':ti,ab,kw OR svm:ti,ab,kw OR 'k-nearest neighbor*':ti,ab,kw OR 'k nearest neighbor*':ti,ab,kw OR 'naive bayes':ti,ab,kw OR 'catboost':ti,ab,kw OR 'ensemble learning':ti,ab,kw OR 'supervised learning':ti,ab,kw OR 'unsupervised learning':ti,ab,kw OR 'boosted tree*':ti,ab,kw OR 'kernel machine*':ti,ab,kw OR radiomic*:ti,ab,kw OR 'radiomics':ti,ab,kw OR 'radiograph* texture*':ti,ab,kw OR 'radiograph* feature*':ti,ab,kw | 1482019 |
| #9 | #7 OR #8 | 2606119 |
| #10 | #3 AND #6 AND #9 | 2869 |

### CINAHL

Starting date: Database creation date

Searched on: November 30, 2025

Results: 558

| **Search** | **Query** |  |
| --- | --- | --- |
| #1 | MH "Fracture Healing" OR MH "Fractures, Ununited+" | 7200 |
| #2 | TI (nonunion* OR "non union*" OR "non-union*" OR nonunited OR "non-united" OR "delayed union*" OR "delayed fracture healing" OR "delayed bone healing" OR malunion* OR "mal-union*" OR pseudoarthros* OR pseudarthros* OR (fractur* N3 heal*) OR "nonhealing fractur*" OR "non-healing fractur*") OR AB (nonunion* OR "non union*" OR "non-union*" OR nonunited OR "non-united" OR "delayed union*" OR "delayed fracture healing" OR "delayed bone healing" OR malunion* OR "mal-union*" OR pseudoarthros* OR pseudarthros* OR (fractur* N3 heal*) OR "nonhealing fractur*" OR "non-healing fractur*") OR SU (nonunion* OR "non union*" OR "non-union*" OR nonunited OR "non-united" OR "delayed union*" OR "delayed fracture healing" OR "delayed bone healing" OR malunion* OR "mal-union*" OR pseudoarthros* OR pseudarthros* OR (fractur* N3 heal*) OR "nonhealing fractur*" OR "non-healing fractur*") | 15529 |
| #3 | #1 or #2 | 15798 |
| #4 | MH "Fractures+" | 73158 |
| #5 | TI (fractur*) OR AB (fractur*) OR SU (fractur*) | 113609 |
| #6 | #4 or #5 | 114049 |
| #7 | MH "Decision Support Techniques+" OR MH "ROC Curve" OR MH "Artificial Intelligence+" | 99572 |
| #8 | TI ( ((predict* OR prognos* OR risk* OR probabilit*) N3 (model* OR score* OR scor* OR index OR tool* OR equation* OR calculator* OR nomogram* OR "prediction rule*" OR "decision rule*" OR "risk stratification")) OR "risk model*" OR "risk prediction" OR "risk assessment" OR "risk scor*" OR "risk score*" OR "risk prediction model*" OR "predictive model*" OR "prediction model*" OR "predictive scor*" OR "prediction tool*" OR "prognostic model*" OR "prognostic index*" OR "prognostic scor*" OR "risk calculator*" OR nomogram* OR "scoring system*" OR "score system*" OR "clinical prediction rule*" OR "clinical decision rule*" OR "multivaria* predict*" OR "multivaria* model*" OR "multivaria* prognos*" OR "machine learning" OR "ml model*" OR "ml-based" OR "ai-based" OR "ai-assisted" OR "artificial intelligence" OR "deep learning" OR "neural network*" OR "convolutional neural network*" OR cnn OR "recurrent neural network*" OR rnn OR "random forest*" OR "decision tree*" OR "gradient boosting" OR xgboost OR "support vector machine*" OR svm OR "k-nearest neighbor*" OR "k nearest neighbor*" OR "naive bayes" OR "catboost" OR "ensemble learning" OR "supervised learning" OR "unsupervised learning" OR "boosted tree*" OR "kernel machine*" OR radiomic* OR "radiomics" OR "radiograph* texture*" OR "radiograph* feature*" ) OR AB ( ((predict* OR prognos* OR risk* OR probabilit*) N3 (model* OR score* OR scor* OR index OR tool* OR equation* OR calculator* OR nomogram* OR "prediction rule*" OR "decision rule*" OR "risk stratification")) OR "risk model*" OR "risk prediction" OR "risk assessment" OR "risk scor*" OR "risk score*" OR "risk prediction model*" OR "predictive model*" OR "prediction model*" OR "predictive scor*" OR "prediction tool*" OR "prognostic model*" OR "prognostic index*" OR "prognostic scor*" OR "risk calculator*" OR nomogram* OR "scoring system*" OR "score system*" OR "clinical prediction rule*" OR "clinical decision rule*" OR "multivaria* predict*" OR "multivaria* model*" OR "multivaria* prognos*" OR "machine learning" OR "ml model*" OR "ml-based" OR "ai-based" OR "ai-assisted" OR "artificial intelligence" OR "deep learning" OR "neural network*" OR "convolutional neural network*" OR cnn OR "recurrent neural network*" OR rnn OR "random forest*" OR "decision tree*" OR "gradient boosting" OR xgboost OR "support vector machine*" OR svm OR "k-nearest neighbor*" OR "k nearest neighbor*" OR "naive bayes" OR "catboost" OR "ensemble learning" OR "supervised learning" OR "unsupervised learning" OR "boosted tree*" OR "kernel machine*" OR radiomic* OR "radiomics" OR "radiograph* texture*" OR "radiograph* feature*" ) | 224712 |
| #9 | #7 or #8 | 289193 |
| #10 | #3 and #6 and #9 | 558 |

### SinoMed

Starting date: Database creation date

Searched on: November 30, 2025

Results: 397

| **Search** | **Query** | **Results** |
| --- | --- | --- |
| #1 | (("骨折, 连接错位"[不加权:扩展]) OR "假关节"[不加权:扩展]) OR "骨折愈合"[不加权:扩展] | 13708 |
| #2 | "骨折不愈合"[标题] OR "骨折不愈合"[关键词] OR "骨折未愈合"[标题] OR "骨折未愈合"[关键词] OR "骨不连"[标题] OR "骨不连"[关键词] OR "骨不愈合"[标题] OR "骨不愈合"[关键词] OR "骨性不连"[标题] OR "骨性不连"[关键词] OR "不连"[标题] OR "不连"[关键词] OR "不愈合"[标题] OR "不愈合"[关键词] OR "延迟愈合"[标题] OR "延迟愈合"[关键词] OR "延迟骨折愈合"[标题] OR "延迟骨折愈合"[关键词] OR "骨折延迟愈合"[标题] OR "骨折延迟愈合"[关键词] OR "迟缓愈合"[标题] OR "迟缓愈合"[关键词] OR "愈合延迟"[标题] OR "愈合延迟"[关键词] OR "畸形愈合"[标题] OR "畸形愈合"[关键词] OR "骨折畸形愈合"[标题] OR "骨折畸形愈合"[关键词] OR "假关节"[标题] OR "假关节"[关键词] OR "假性关节"[标题] OR "假性关节"[关键词] OR "nonunion"[标题] OR "nonunion"[关键词] OR "non-union"[标题] OR "non-union"[关键词] OR "delayed union"[标题] OR "delayed union"[关键词] OR "malunion"[标题] OR "malunion"[关键词] OR "pseudoarthrosis"[标题] OR "pseudoarthrosis"[关键词] OR "pseudarthrosis"[标题] OR "pseudarthrosis"[关键词] OR "fracture healing"[标题] OR "fracture healing"[关键词] | 7569 |
| #3 | "骨折"[不加权:扩展] OR "骨折"[标题] OR "骨折"[关键词] OR "fracture"[标题] OR "fracture"[关键词] OR "fractures"[标题] OR "fractures"[关键词] OR "bone fracture"[标题] OR "bone fracture"[关键词] OR "bone fractures"[标题] OR "bone fractures"[关键词] | 274027 |
| #4 | "风险评估"[摘要] OR "风险预测"[摘要] OR "风险模型"[摘要] OR "风险预测模型"[摘要] OR "预测模型"[摘要] OR "预后模型"[摘要] OR "预后预测"[摘要] OR "评分系统"[摘要] OR "评分体系"[摘要] OR "列线图"[摘要] OR "Nomogram"[摘要] OR "机器学习"[摘要] OR "深度学习"[摘要] OR "随机森林"[摘要] OR "支持向量机"[摘要] OR "神经网络"[摘要] OR "Logistic回归"[摘要] OR "Logistic模型"[摘要] OR "Cox回归"[摘要] OR "ROC曲线"[摘要] OR "AUC"[摘要] OR "C指数"[摘要] | 350026 |
| #5 | (#1 OR #2) AND #3 AND #4 | 397 |

### CNKI

Starting date: Database creation date

Searched on: November 30, 2025

Results: 478

| **Search** | **Query** | **Results** |
| --- | --- | --- |
| S1 | SU%=('骨折不愈合' + '骨折未愈合' + '骨折不连' + '骨不连' + '骨折延迟愈合' + '延迟骨折愈合' + '骨折畸形愈合' + '假关节' + 'nonunion' + 'non-union' + 'delayed union' + 'malunion' + 'pseudoarthrosis' + 'pseudarthrosis') AND TKA%=('风险评估' + '风险预测' + '风险模型' + '风险预测模型' + '预测模型' + '预后模型' + '预后预测' + '评分系统' + '评分体系' + '列线图' + 'Nomogram' + '机器学习' + '深度学习' + '随机森林' + '支持向量机' + '神经网络' + 'Logistic回归' + 'Logistic模型' + 'Cox回归' + 'ROC曲线' + 'AUC' + 'C指数') | 478 |

# Supplementary Figures

## Figure S1. calibration reporting rate


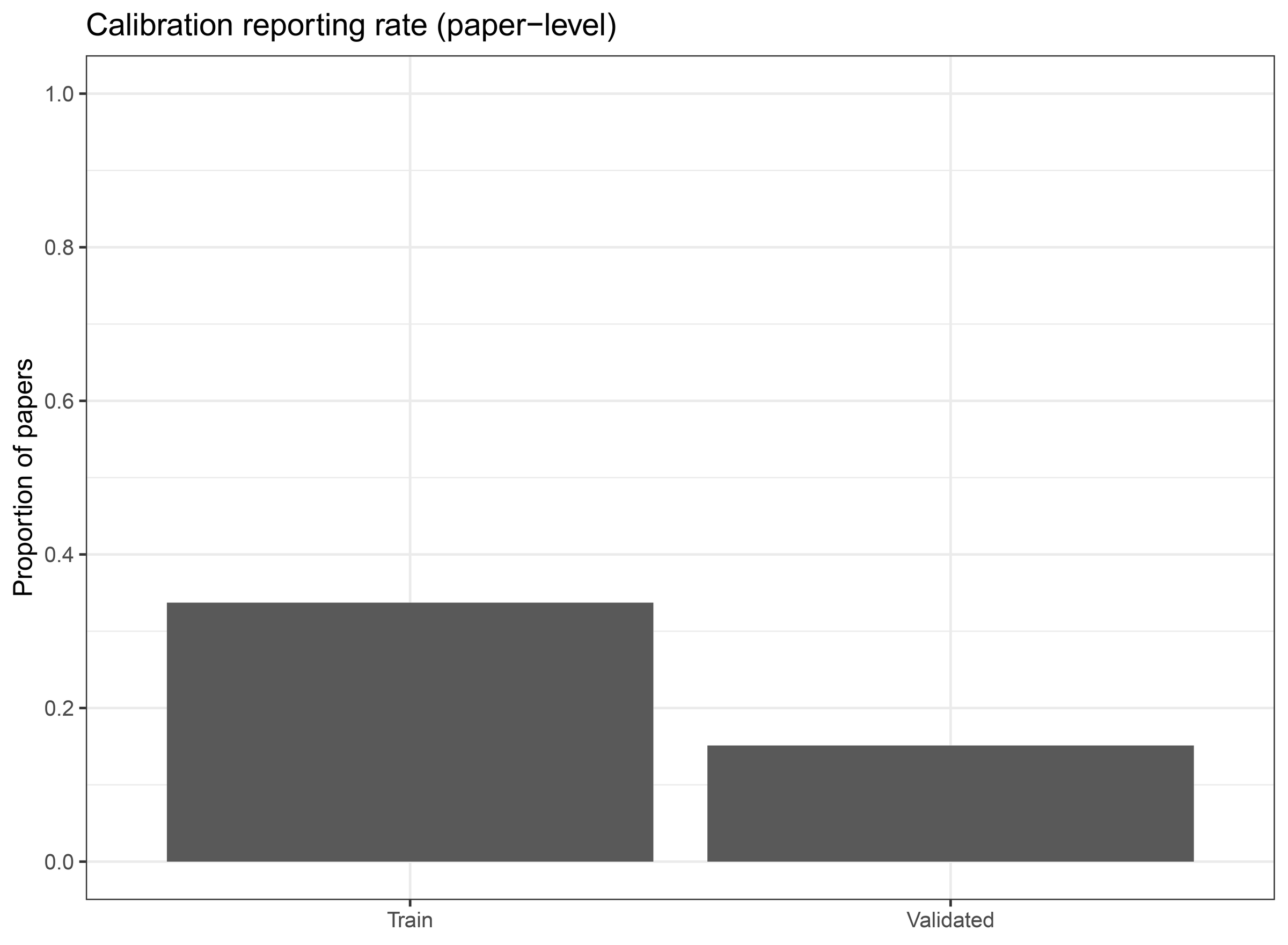


Figure S1. calibration reporting rate.

## Figure S2. optimism train vs validated


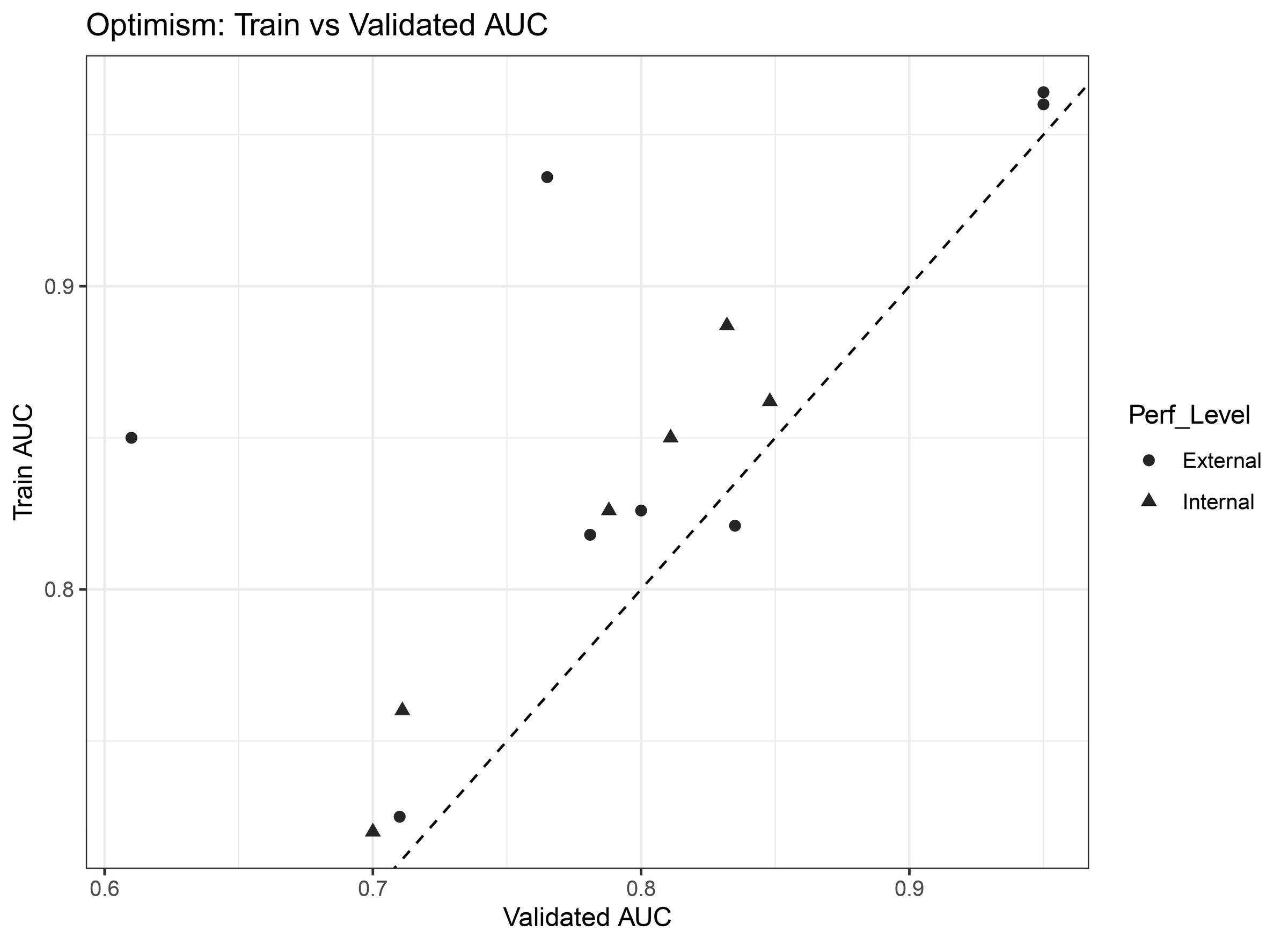


Figure S2. optimism train vs validated.

## Figure S3. Forest plot of pooled AUC for apparent (development) performance


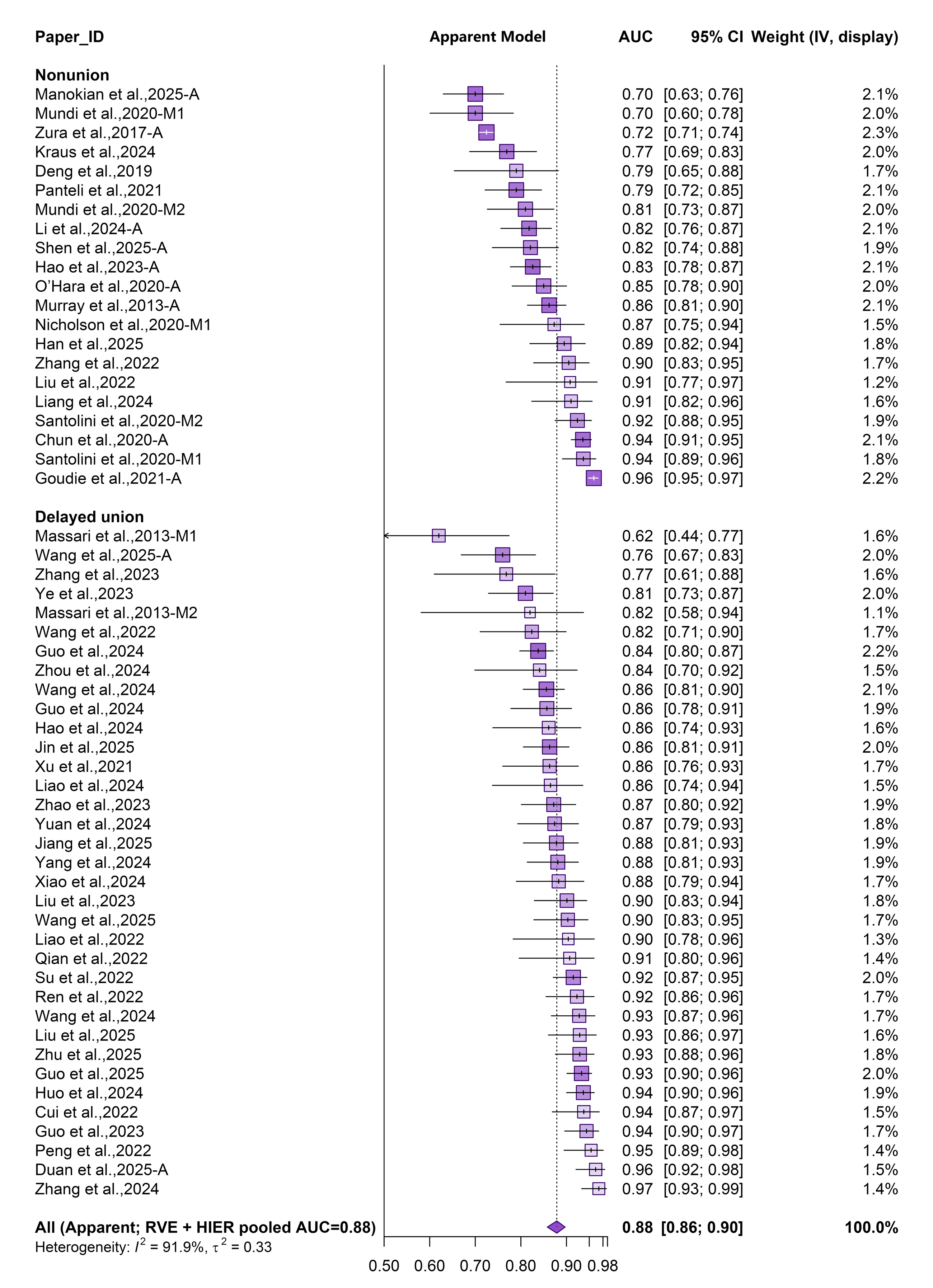


Figure S3. Forest plot of pooled AUC for apparent (development) performance.

## Figure S4. Leave-one-out sensitivity analyses for pooled AUC estimates


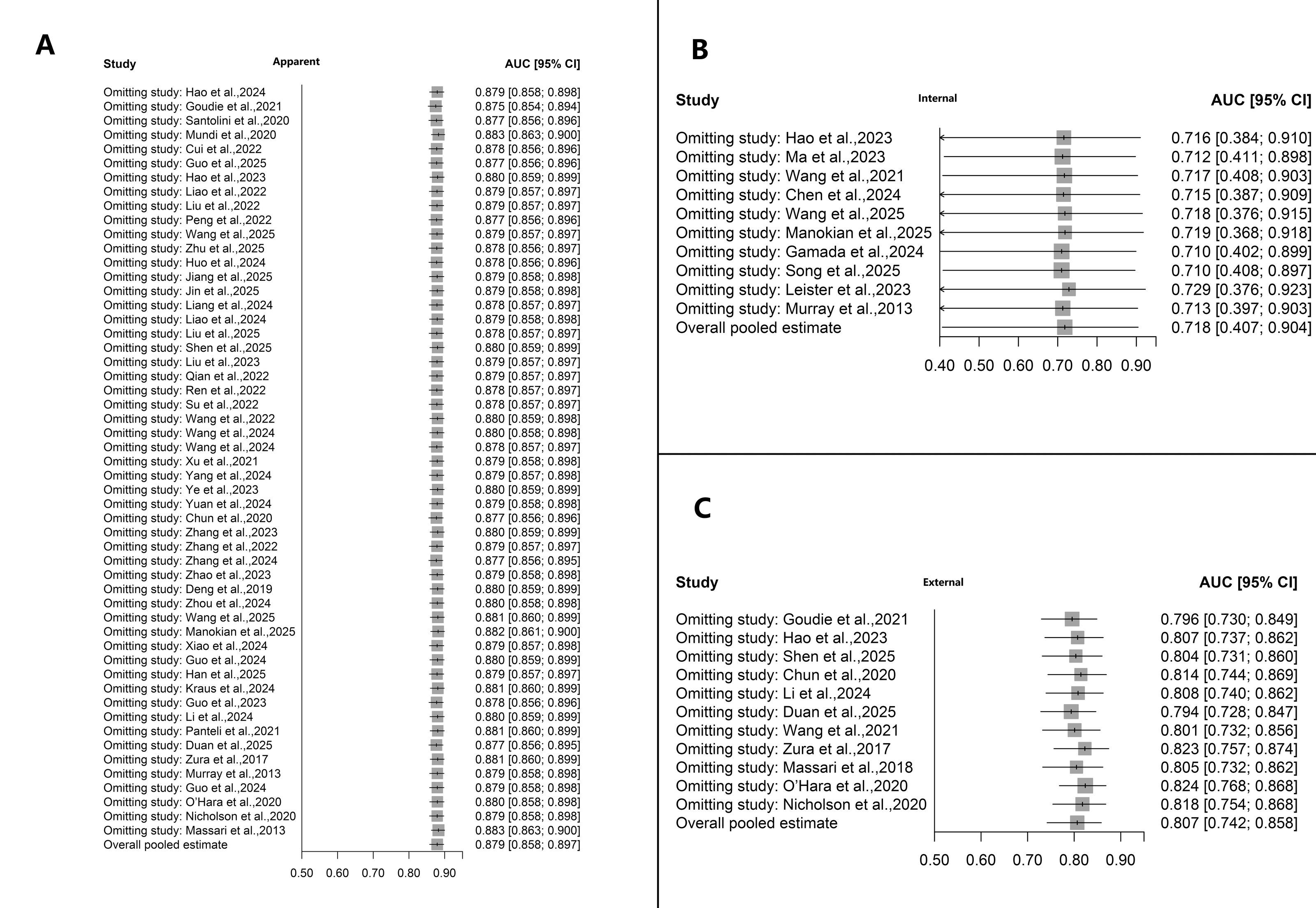


Figure S4. Leave-one-out sensitivity analyses for pooled AUC estimates.

## Figure S5. Funnel plot for small-study effects in apparent (development) performance


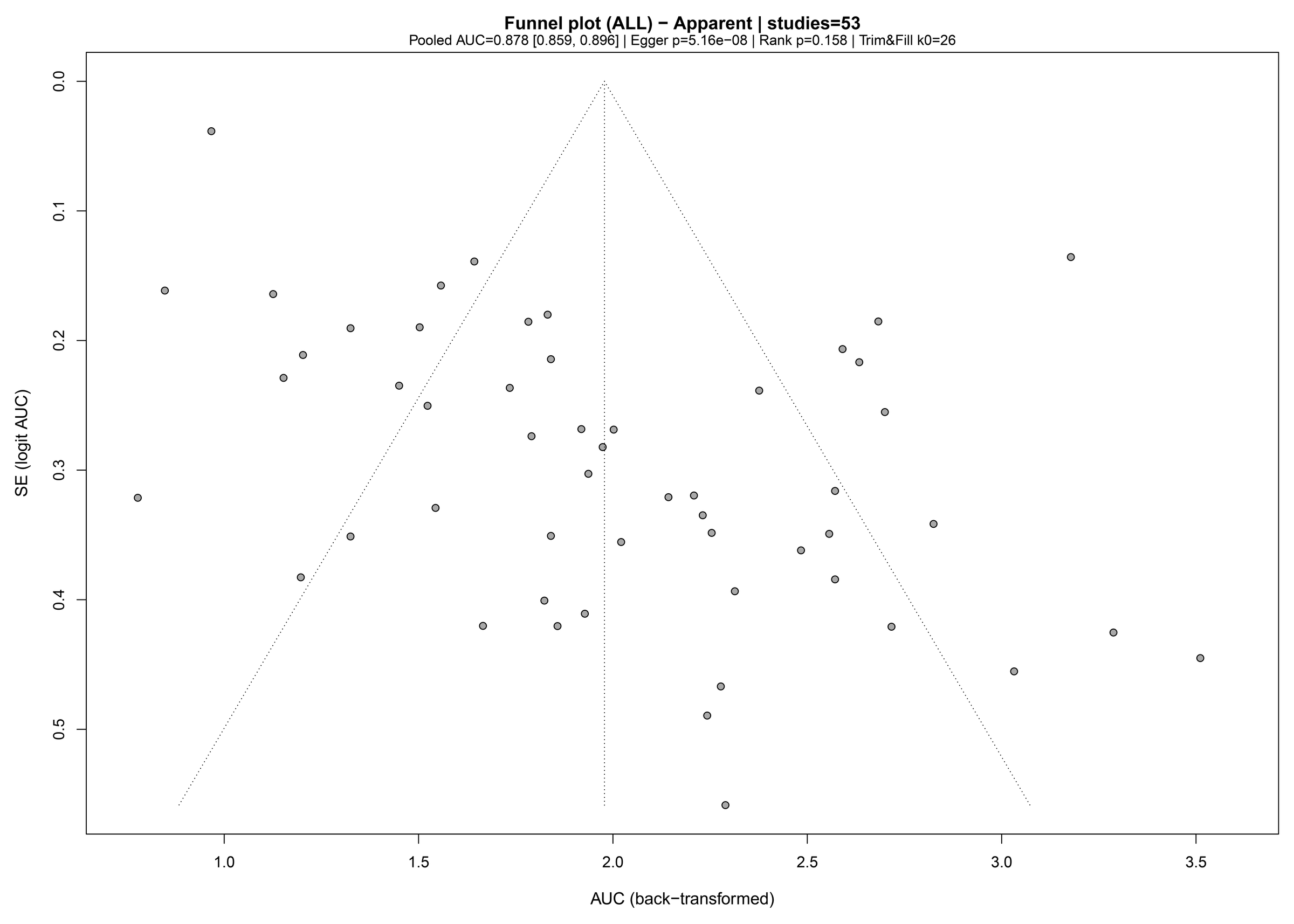


Figure S5. Funnel plot for small-study effects in apparent (development) performance.

## Figure S6. Funnel plot for small-study effects in internal validation


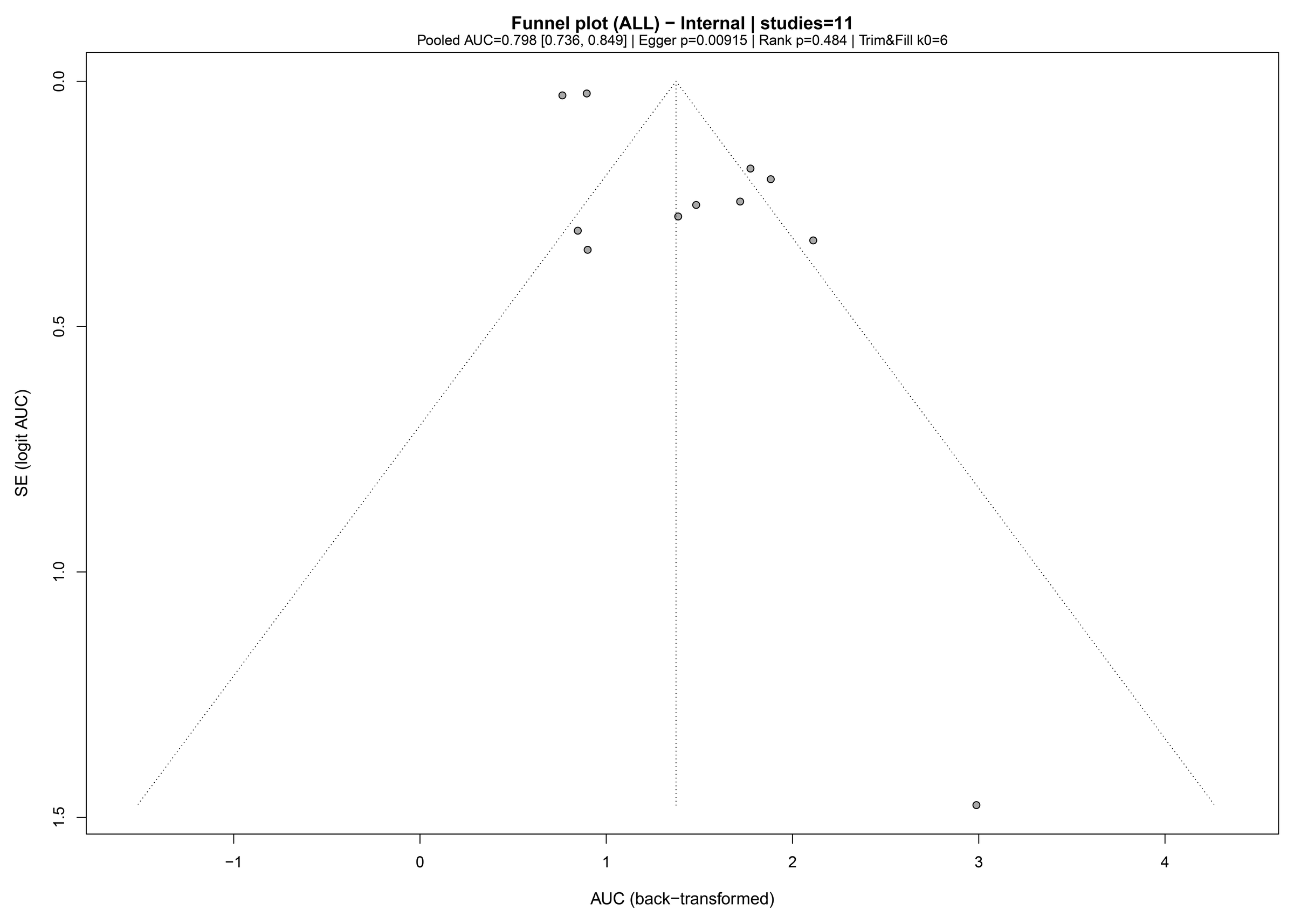


Figure S6. Funnel plot for small-study effects in internal validation.

## Figure S7. Funnel plot for small-study effects in external validation


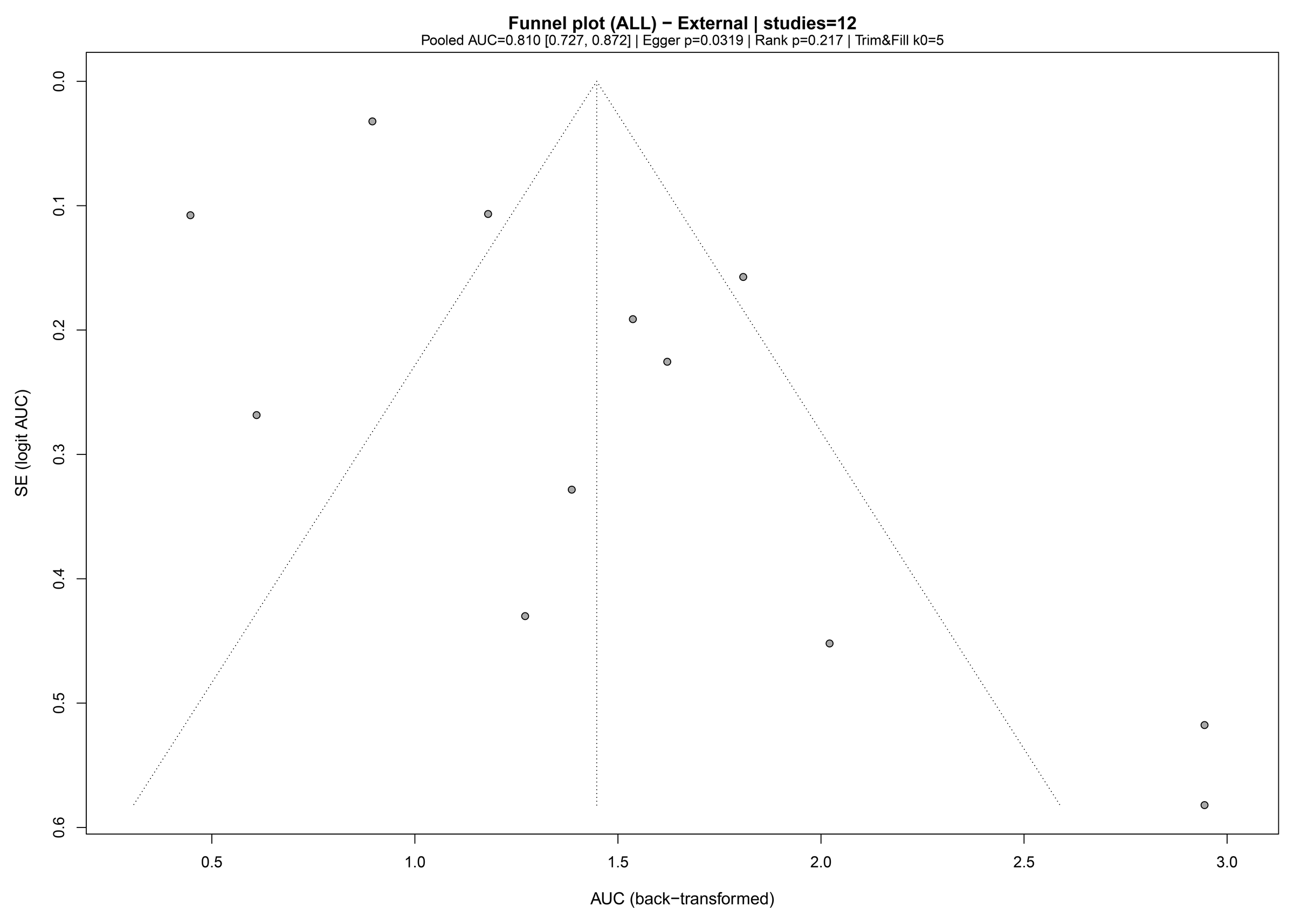


Figure S7. Funnel plot for small-study effects in external validation.

## Table S8. Traceability lists for included studies cited in the Results and Discussion (Author, year).

| Category | Count / ID type | Supporting Study_ID_base or Study_ID list |
| --- | --- | --- |
| Study-level: Country — China | n=56 | Shen et al.,2025, Chen et al.,2024, Chun et al.,2020, Deng et al.,2019, Guo et al.,2024, Guo et al.,2023, Guo et al.,2024, Hao et al.,2024, He et al.,2024, Huo et al.,2024, Jiang et al.,2025, Jin et al.,2025, Liang et al.,2024, Liao et al.,2024 Liu et al.,2025, Liu et al.,2023, Liu et al.,2025, Ma et al.,2023, Qian et al.,2022, Ren et al.,2022, Su et al.,2022, Wang et al.,2022, Wang et al.,2024, Wang et al.,2021, Wang et al.,2024, Xu et al.,2021, Yang et al.,2020, Yang et al.,2024 Ye et al.,2023, Yuan et al.,2024, Zhang et al.,2023, Zhang et al.,2022, Zhang et al.,2024, Zhao et al.,2023, Zheng et al.,2024, Zhou et al.,2024, Wang et al.,2025, Tang et al.,2025, Song et al.,2025, Zhu et al.,2025, Wu et al.,2025, Xiao et al.,2024 Han et al.,2025, Li et al.,2024, Duan et al.,2025, Wang et al.,2021, Zhu et al.,2021, Chen et al.,2025, Cui et al.,2022, Guo et al.,2025, Hao et al.,2023, Liao et al.,2022, Liu et al.,2022, Peng et al.,2022, Wang et al.,2025, Zhu et al.,2025 |
| Study-level: Country — UK | n=5 | Panteli et al.,2021, Murray et al.,2013, Nicholson et al.,2020, Ramamurthy et al.,2007, Goudie et al.,2021 |
| Study-level: Country — USA | n=4 | Kraus et al.,2024, Zura et al.,2017, Maceroli et al.,2017, Zura et al.,2017 |
| Study-level: Country — Multinational collaborations | n=4 | Leister et al.,2023, O’Hara et al.,2020, Santolini et al.,2020, Mundi et al.,2020 |
| Study-level: Design — Retrospective cohort | n=62 | Shen et al.,2025, Chen et al.,2024, Chun et al.,2020, Deng et al.,2019, Guo et al.,2024, Guo et al.,2023, Guo et al.,2024, Hao et al.,2024, He et al.,2024, Jiang et al.,2025, Jin et al.,2025, Liang et al.,2024, Liao et al.,2024, Liu et al.,2025 Liu et al.,2023, Liu et al.,2025, Ma et al.,2023, Qian et al.,2022, Ren et al.,2022, Su et al.,2022, Wang et al.,2022, Wang et al.,2024, Wang et al.,2021, Xu et al.,2021, Yang et al.,2020, Yang et al.,2024, Ye et al.,2023, Zhang et al.,2023 Zhang et al.,2022, Zhang et al.,2024, Zheng et al.,2024, Zhou et al.,2024, Wang et al.,2025, Manokian et al.,2025, Tang et al.,2025, Braun et al.,2025, Gamada et al.,2024, Zhu et al.,2025, Wu et al.,2025, Xiao et al.,2024, Leister et al.,2023, Han et al.,2025 Hsu et al.,2016, Kraus et al.,2024, Li et al.,2024, Suter et al.,2024, Panteli et al.,2021, Duan et al.,2025, Wang et al.,2021, Murray et al.,2013, Zhu et al.,2021, Maceroli et al.,2017, Massari et al.,2013, Ramamurthy et al.,2007, Goudie et al.,2021, Mundi et al.,2020 Cui et al.,2022, Guo et al.,2025, Hao et al.,2023, Liao et al.,2022, Liu et al.,2022, Zhu et al.,2025 |
| Study-level: Design — Prospective cohort | n=6 | Huo et al.,2024, Song et al.,2025, Haubruck et al.,2018, Massari et al.,2018, Nicholson et al.,2020, Wang et al.,2025 |
| Study-level: Design — Case–control | n=6 | Wang et al.,2024, Yuan et al.,2024, Zhao et al.,2023, Santolini et al.,2020, Chen et al.,2025, Peng et al.,2022 |
| Study-level: Design — Database study | n=2 | Zura et al.,2017, Zura et al.,2017 |
| Study-level: Design — Secondary analysis of RCT | n=1 | O’Hara et al.,2020 |
| Study-level: Studies contributing >1 model | n=17 | Shen et al.,2025, Chun et al.,2020, Wang et al.,2025, Manokian et al.,2025, Li et al.,2024, Suter et al.,2024, Duan et al.,2025, Zura et al.,2017, Murray et al.,2013, O’Hara et al.,2020, Nicholson et al.,2020, Zura et al.,2017, Massari et al.,2013, Goudie et al.,2021 Santolini et al.,2020, Mundi et al.,2020, Hao et al.,2023 |
| Study-level: Study contributing 18 models | K=1 | Zura et al.,2017 |
| Model-level: Validation tier — Apparent | k=56 | Shen et al.,2025 (B), Chun et al.,2020 (B), Deng et al.,2019, Guo et al.,2024 (A), Guo et al.,2023, Guo et al.,2024, Hao et al.,2024, Huo et al.,2024, Jiang et al.,2025, Jin et al.,2025, Liang et al.,2024, Liao et al.,2024 Liu et al.,2025, Liu et al.,2023, Qian et al.,2022, Ren et al.,2022, Su et al.,2022, Wang et al.,2022, Wang et al.,2024, Wang et al.,2024, Xu et al.,2021, Yang et al.,2024, Ye et al.,2023, Yuan et al.,2024 Zhang et al.,2023, Zhang et al.,2022, Zhang et al.,2024, Zhao et al.,2023, Zhou et al.,2024, Wang et al.,2025 (B), Manokian et al.,2025 (B), Xiao et al.,2024, Han et al.,2025, Kraus et al.,2024, Li et al.,2024 (B), Panteli et al.,2021 (B) Duan et al.,2025 (B), Zura et al.,2017 (B), Murray et al.,2013 (B), O’Hara et al.,2020 (B), Nicholson et al.,2020 (A), Massari et al.,2013 (A), Massari et al.,2013 (B), Goudie et al.,2021 (B), Santolini et al.,2020 (A), Santolini et al.,2020 (B), Mundi et al.,2020 (A), Mundi et al.,2020 (B) Cui et al.,2022, Guo et al.,2025, Hao et al.,2023 (C), Liao et al.,2022, Liu et al.,2022, Peng et al.,2022, Wang et al.,2025, Zhu et al.,2025 |
| Model-level: Validation tier — Internal | k=28 | Chen et al.,2024, Ma et al.,2023, Wang et al.,2021, Wang et al.,2025 (A), Manokian et al.,2025 (A), Gamada et al.,2024, Song et al.,2025, Leister et al.,2023 (A), Murray et al.,2013 (A), Zura et al.,2017 (A), Zura et al.,2017 (B), Zura et al.,2017 (C) Zura et al.,2017 (D), Zura et al.,2017 (E), Zura et al.,2017 (F), Zura et al.,2017 (G), Zura et al.,2017 (H), Zura et al.,2017 (I), Zura et al.,2017 (J), Zura et al.,2017 (K), Zura et al.,2017 (L), Zura et al.,2017 (M), Zura et al.,2017 (N), Zura et al.,2017 (O) Zura et al.,2017 (P), Zura et al.,2017 (Q), Zura et al.,2017 (R), Hao et al.,2023 (B) |
| Model-level: Validation tier — External | k=13 | Shen et al.,2025 (A), Chun et al.,2020 (A), Li et al.,2024 (A), Suter et al.,2024 (A), Suter et al.,2024 (B), Duan et al.,2025 (A), Wang et al.,2021, Zura et al.,2017 (A), Massari et al.,2018, O’Hara et al.,2020 (A), Nicholson et al.,2020 (B), Goudie et al.,2021 (A) Hao et al.,2023 (A) |
| Model-level: Target outcome — Nonunion | k=59 | Shen et al.,2025 (A), Shen et al.,2025 (B), Chen et al.,2024, Chun et al.,2020 (A), Chun et al.,2020 (B), Deng et al.,2019, Liang et al.,2024, Ma et al.,2023, Wang et al.,2021, Zhang et al.,2022, Manokian et al.,2025 (A), Manokian et al.,2025 (B) Gamada et al.,2024, Leister et al.,2023 (A), Han et al.,2025, Kraus et al.,2024, Li et al.,2024 (A), Li et al.,2024 (B), Suter et al.,2024 (A), Suter et al.,2024 (B), Panteli et al.,2021 (B), Wang et al.,2021, Zura et al.,2017 (A), Zura et al.,2017 (B) Murray et al.,2013 (A), Murray et al.,2013 (B), Massari et al.,2018, O’Hara et al.,2020 (A), O’Hara et al.,2020 (B), Nicholson et al.,2020 (A), Nicholson et al.,2020 (B), Zura et al.,2017 (A), Zura et al.,2017 (B), Zura et al.,2017 (C), Zura et al.,2017 (D), Zura et al.,2017 (E) Zura et al.,2017 (F), Zura et al.,2017 (G), Zura et al.,2017 (H), Zura et al.,2017 (I), Zura et al.,2017 (J), Zura et al.,2017 (K), Zura et al.,2017 (L), Zura et al.,2017 (M), Zura et al.,2017 (N), Zura et al.,2017 (O), Zura et al.,2017 (P), Zura et al.,2017 (Q) Zura et al.,2017 (R), Goudie et al.,2021 (A), Goudie et al.,2021 (B), Santolini et al.,2020 (A), Santolini et al.,2020 (B), Mundi et al.,2020 (A), Mundi et al.,2020 (B), Hao et al.,2023 (A), Hao et al.,2023 (B), Hao et al.,2023 (C), Liu et al.,2022 |
| Model-level: Target outcome — Delayed union | k=38 | Guo et al.,2024 (A), Guo et al.,2023, Guo et al.,2024, Hao et al.,2024, Huo et al.,2024, Jiang et al.,2025, Jin et al.,2025, Liao et al.,2024, Liu et al.,2025, Liu et al.,2023, Qian et al.,2022, Ren et al.,2022 Su et al.,2022, Wang et al.,2022, Wang et al.,2024, Wang et al.,2024, Xu et al.,2021, Yang et al.,2024, Ye et al.,2023, Yuan et al.,2024, Zhang et al.,2023, Zhang et al.,2024, Zhao et al.,2023, Zhou et al.,2024 Wang et al.,2025 (A), Wang et al.,2025 (B), Song et al.,2025, Xiao et al.,2024, Duan et al.,2025 (A), Duan et al.,2025 (B), Massari et al.,2013 (A), Massari et al.,2013 (B), Cui et al.,2022, Guo et al.,2025, Liao et al.,2022, Peng et al.,2022 Wang et al.,2025, Zhu et al.,2025 |
| Model-level: Fracture site — Lower limb | k=54 | Chen et al.,2024, Chun et al.,2020 (A), Chun et al.,2020 (B), Deng et al.,2019, Jin et al.,2025, Liang et al.,2024, Liu et al.,2023, Ma et al.,2023, Qian et al.,2022, Ren et al.,2022, Su et al.,2022, Wang et al.,2022 Wang et al.,2021, Xu et al.,2021, Ye et al.,2023, Yuan et al.,2024, Zhang et al.,2022, Zhang et al.,2024, Zhao et al.,2023, Zhou et al.,2024, Wang et al.,2025 (A), Wang et al.,2025 (B), Kraus et al.,2024, Li et al.,2024 (A) Li et al.,2024 (B), Panteli et al.,2021 (B), Wang et al.,2021, Massari et al.,2018, O’Hara et al.,2020 (A), O’Hara et al.,2020 (B), Zura et al.,2017 (A), Zura et al.,2017 (C), Zura et al.,2017 (F), Zura et al.,2017 (H), Zura et al.,2017 (N), Zura et al.,2017 (O) Zura et al.,2017 (P), Zura et al.,2017 (Q), Zura et al.,2017 (R), Massari et al.,2013 (A), Massari et al.,2013 (B), Santolini et al.,2020 (A), Santolini et al.,2020 (B), Mundi et al.,2020 (A), Mundi et al.,2020 (B), Cui et al.,2022, Guo et al.,2025, Hao et al.,2023 (A) Hao et al.,2023 (B), Hao et al.,2023 (C), Liao et al.,2022, Liu et al.,2022, Peng et al.,2022, Wang et al.,2025 |
| Model-level: Prediction horizon group — <=3mo | k=21 | Guo et al.,2024 (A), Guo et al.,2024, Hao et al.,2024, Jiang et al.,2025, Jin et al.,2025, Liu et al.,2025, Ren et al.,2022, Su et al.,2022, Wang et al.,2022, Wang et al.,2024, Wang et al.,2024, Yang et al.,2024 Yuan et al.,2024, Zhang et al.,2023, Song et al.,2025, Suter et al.,2024 (A), Suter et al.,2024 (B), Cui et al.,2022, Liu et al.,2022, Peng et al.,2022, Zhu et al.,2025 |
| Model-level: Prediction horizon group — >3-6mo | k=22 | Guo et al.,2023, Huo et al.,2024, Liu et al.,2023, Qian et al.,2022, Zhao et al.,2023, Zhou et al.,2024, Manokian et al.,2025 (A), Manokian et al.,2025 (B), Gamada et al.,2024, Xiao et al.,2024, Leister et al.,2023 (A), Han et al.,2025 Duan et al.,2025 (A), Duan et al.,2025 (B), Murray et al.,2013 (A), Murray et al.,2013 (B), Nicholson et al.,2020 (A), Nicholson et al.,2020 (B), Goudie et al.,2021 (A), Goudie et al.,2021 (B), Guo et al.,2025, Wang et al.,2025 |
| Model-level: Prediction horizon group — >6-12mo | k=52 | Shen et al.,2025 (A), Shen et al.,2025 (B), Chen et al.,2024, Chun et al.,2020 (A), Chun et al.,2020 (B), Deng et al.,2019, Liang et al.,2024, Liao et al.,2024, Ma et al.,2023, Wang et al.,2021, Xu et al.,2021, Ye et al.,2023 Zhang et al.,2022, Wang et al.,2025 (A), Wang et al.,2025 (B), Li et al.,2024 (A), Li et al.,2024 (B), Panteli et al.,2021 (B), Wang et al.,2021, Zura et al.,2017 (A), Zura et al.,2017 (B), Massari et al.,2018, O’Hara et al.,2020 (A), O’Hara et al.,2020 (B) Zura et al.,2017 (A), Zura et al.,2017 (B), Zura et al.,2017 (C), Zura et al.,2017 (D), Zura et al.,2017 (E), Zura et al.,2017 (F), Zura et al.,2017 (G), Zura et al.,2017 (H), Zura et al.,2017 (I), Zura et al.,2017 (J), Zura et al.,2017 (K), Zura et al.,2017 (L) Zura et al.,2017 (M), Zura et al.,2017 (N), Zura et al.,2017 (O), Zura et al.,2017 (P), Zura et al.,2017 (Q), Zura et al.,2017 (R), Massari et al.,2013 (A), Massari et al.,2013 (B), Santolini et al.,2020 (A), Santolini et al.,2020 (B), Mundi et al.,2020 (A), Mundi et al.,2020 (B) Hao et al.,2023 (A), Hao et al.,2023 (B), Hao et al.,2023 (C), Liao et al.,2022 |
| Model-level: Prediction horizon group — >12-24mo | k=1 | Kraus et al.,2024 |
| Model-level: Prediction horizon group — NR | k=1 | Zhang et al.,2024 |
| Model-level: Outcome ascertainment — Clinical + Imaging | k=0 |  |
| Model-level: Outcome ascertainment — Claims/Registry (proxy) | k=0 |  |
| Model-level: Predictor domain — Serum/Blood biomarkers | k=27 | Deng et al.,2019, Hao et al.,2024, Jiang et al.,2025, Jin et al.,2025, Liang et al.,2024, Liao et al.,2024, Liu et al.,2025, Liu et al.,2023, Qian et al.,2022, Wang et al.,2022, Wang et al.,2024, Wang et al.,2024 Xu et al.,2021, Zhang et al.,2023, Zhang et al.,2022, Zhang et al.,2024, Zhao et al.,2023, Wang et al.,2025 (A), Wang et al.,2025 (B), Xiao et al.,2024, Duan et al.,2025 (A), Duan et al.,2025 (B), Cui et al.,2022, Liao et al.,2022 Liu et al.,2022, Wang et al.,2025, Zhu et al.,2025 |
| Model-level: Calibration reported in development | k=25 | Shen et al.,2025 (A), Shen et al.,2025 (B), Chen et al.,2024, Chun et al.,2020 (A), Chun et al.,2020 (B), Guo et al.,2024 (A), Wang et al.,2025 (A), Wang et al.,2025 (B), Manokian et al.,2025 (A), Manokian et al.,2025 (B), Song et al.,2025, Li et al.,2024 (A) Li et al.,2024 (B), Panteli et al.,2021 (B), Murray et al.,2013 (A), Murray et al.,2013 (B), O’Hara et al.,2020 (A), O’Hara et al.,2020 (B), Goudie et al.,2021 (A), Goudie et al.,2021 (B), Santolini et al.,2020 (A), Santolini et al.,2020 (B), Hao et al.,2023 (A), Hao et al.,2023 (B) Hao et al.,2023 (C) |
| Model-level: Calibration reported on validation data | k=10 | Shen et al.,2025 (A), Chun et al.,2020 (A), Ma et al.,2023, Wang et al.,2021, Wang et al.,2025 (A), Li et al.,2024 (A), Wang et al.,2021, O’Hara et al.,2020 (A), Hao et al.,2023 (A), Hao et al.,2023 (B) |
| Model-level: Missing data handling — Not reported (NR) | k=78 | Shen et al.,2025 (A), Shen et al.,2025 (B), Chen et al.,2024, Chun et al.,2020 (A), Chun et al.,2020 (B), Deng et al.,2019, Guo et al.,2024 (A), Guo et al.,2023, Guo et al.,2024, Hao et al.,2024, Huo et al.,2024, Jiang et al.,2025 Jin et al.,2025, Liang et al.,2024, Liao et al.,2024, Liu et al.,2025, Liu et al.,2023, Ma et al.,2023, Qian et al.,2022, Ren et al.,2022, Su et al.,2022, Wang et al.,2022, Wang et al.,2024, Wang et al.,2021 Wang et al.,2024, Xu et al.,2021, Yang et al.,2024, Ye et al.,2023, Yuan et al.,2024, Zhang et al.,2023, Zhang et al.,2022, Zhang et al.,2024, Zhao et al.,2023, Zhou et al.,2024, Wang et al.,2025 (A), Wang et al.,2025 (B) Song et al.,2025, Xiao et al.,2024, Leister et al.,2023 (A), Han et al.,2025, Li et al.,2024 (A), Li et al.,2024 (B), Panteli et al.,2021 (B), Wang et al.,2021, Zura et al.,2017 (A), Zura et al.,2017 (B), O’Hara et al.,2020 (A), O’Hara et al.,2020 (B) Nicholson et al.,2020 (A), Nicholson et al.,2020 (B), Zura et al.,2017 (A), Zura et al.,2017 (B), Zura et al.,2017 (C), Zura et al.,2017 (D), Zura et al.,2017 (E), Zura et al.,2017 (F), Zura et al.,2017 (G), Zura et al.,2017 (H), Zura et al.,2017 (I), Zura et al.,2017 (J) Zura et al.,2017 (K), Zura et al.,2017 (L), Zura et al.,2017 (M), Zura et al.,2017 (N), Zura et al.,2017 (O), Zura et al.,2017 (P), Zura et al.,2017 (Q), Zura et al.,2017 (R), Cui et al.,2022, Guo et al.,2025, Hao et al.,2023 (A), Hao et al.,2023 (B) Hao et al.,2023 (C), Liao et al.,2022, Liu et al.,2022, Peng et al.,2022, Wang et al.,2025, Zhu et al.,2025 |
| Model-level: Missing data handling — Complete-case analysis | k=17 | Manokian et al.,2025 (A), Manokian et al.,2025 (B), Gamada et al.,2024, Kraus et al.,2024, Suter et al.,2024 (A), Suter et al.,2024 (B), Duan et al.,2025 (A), Duan et al.,2025 (B), Murray et al.,2013 (A), Murray et al.,2013 (B), Massari et al.,2018, Massari et al.,2013 (A) Massari et al.,2013 (B), Santolini et al.,2020 (A), Santolini et al.,2020 (B), Mundi et al.,2020 (A), Mundi et al.,2020 (B) |
| Model-level: Missing data handling — Multiple imputation | k=2 | Goudie et al.,2021 (A), Goudie et al.,2021 (B) |
| Model-level: EPV <10 (among models with calculable EPV) | k=20 | Chun et al.,2020 (A), Chun et al.,2020 (B), Deng et al.,2019, Guo et al.,2024 (A), Liu et al.,2023, Ma et al.,2023, Ren et al.,2022, Wang et al.,2022, Wang et al.,2021, Zhang et al.,2022, Zhang et al.,2024, Wang et al.,2021 O’Hara et al.,2020 (A), O’Hara et al.,2020 (B), Nicholson et al.,2020 (A), Zura et al.,2017 (M), Massari et al.,2013 (A), Massari et al.,2013 (B), Cui et al.,2022, Liu et al.,2022 |
| Model-level: Paired train–validated AUC comparisons | k=12 | Shen et al.,2025 (A), Chun et al.,2020 (A), Wang et al.,2025 (A), Manokian et al.,2025 (A), Li et al.,2024 (A), Duan et al.,2025 (A), Zura et al.,2017 (A), Murray et al.,2013 (A), O’Hara et al.,2020 (A), Goudie et al.,2021 (A), Hao et al.,2023 (A), Hao et al.,2023 (B) |
| Model-level: Internal validation method includes split-sample | k=25 | Ma et al.,2023, Wang et al.,2021, Wang et al.,2025 (A), Manokian et al.,2025 (A), Leister et al.,2023 (A), Murray et al.,2013 (A), Zura et al.,2017 (A), Zura et al.,2017 (B), Zura et al.,2017 (C), Zura et al.,2017 (D), Zura et al.,2017 (E), Zura et al.,2017 (F) Zura et al.,2017 (G), Zura et al.,2017 (H), Zura et al.,2017 (I), Zura et al.,2017 (J), Zura et al.,2017 (K), Zura et al.,2017 (L), Zura et al.,2017 (M), Zura et al.,2017 (N), Zura et al.,2017 (O), Zura et al.,2017 (P), Zura et al.,2017 (Q), Zura et al.,2017 (R) Hao et al.,2023 (B) |
| Model-level: Predictor selection includes univariable screening and/or stepwise | k=51 | Shen et al.,2025 (A), Chen et al.,2024, Chun et al.,2020 (A), Chun et al.,2020 (B), Liu et al.,2023, Ma et al.,2023, Qian et al.,2022, Wang et al.,2021, Yuan et al.,2024, Wang et al.,2025 (A), Wang et al.,2025 (B), Manokian et al.,2025 (A) Manokian et al.,2025 (B), Han et al.,2025, Kraus et al.,2024, Li et al.,2024 (A), Panteli et al.,2021 (B), Duan et al.,2025 (A), Duan et al.,2025 (B), Wang et al.,2021, Zura et al.,2017 (A), Zura et al.,2017 (B), Murray et al.,2013 (A), Murray et al.,2013 (B) O’Hara et al.,2020 (A), O’Hara et al.,2020 (B), Nicholson et al.,2020 (A), Zura et al.,2017 (A), Zura et al.,2017 (B), Zura et al.,2017 (C), Zura et al.,2017 (D), Zura et al.,2017 (E), Zura et al.,2017 (F), Zura et al.,2017 (G), Zura et al.,2017 (H), Zura et al.,2017 (I) Zura et al.,2017 (J), Zura et al.,2017 (K), Zura et al.,2017 (L), Zura et al.,2017 (M), Zura et al.,2017 (N), Zura et al.,2017 (O), Zura et al.,2017 (P), Zura et al.,2017 (Q), Zura et al.,2017 (R), Goudie et al.,2021 (A), Goudie et al.,2021 (B), Guo et al.,2025 Hao et al.,2023 (A), Hao et al.,2023 (B), Hao et al.,2023 (C) |
